# Genomic variant-identification methods may alter *Mycobacterium tuberculosis* transmission inferences

**DOI:** 10.1099/mgen.0.000418

**Published:** 2020-07-31

**Authors:** Katharine S. Walter, Caroline Colijn, Ted Cohen, Barun Mathema, Qingyun Liu, Jolene Bowers, David M. Engelthaler, Apurva Narechania, Darrin Lemmer, Julio Croda, Jason R. Andrews

**Affiliations:** ^1^​ Division of Infectious Diseases and Geographic Medicine, Stanford University School of Medicine, Stanford, CA, USA; ^2^​ Department of Mathematics, Simon Fraser University, Burnaby, BC, Canada; ^3^​ Department of Epidemiology of Microbial Diseases, Yale School of Public Health, New Haven, CT, USA; ^4^​ Department of Epidemiology, Mailman School of Public Health, Columbia University Medical Center, New York, New York, USA; ^5^​ School of Basic Medical Science of Fudan University, Shanghai, PR China; ^6^​ Translational Genomics Research Institute, Flagstaff, AZ, USA; ^7^​ American Museum of Natural History, New York City, NY, USA; ^8^​ School of Medicine, Federal University of Mato Grosso do Sul, Campo Grande, Brazil; ^9^​ Oswaldo Cruz Foundation, Campo Grande, Brazil; ^10^​ Division of Infectious Diseases and Geographic Medicine, Stanford University School of Medicine, Stanford, CA, USA

**Keywords:** genomic epidemiology, pathogen genomics, transmission, tuberculosis, variant identification

## Abstract

Pathogen genomic data are increasingly used to characterize global and local transmission patterns of important human pathogens and to inform public health interventions. Yet, there is no current consensus on how to measure genomic variation. To test the effect of the variant-identification approach on transmission inferences for *Mycobacterium tuberculosis,* we conducted an experiment in which five genomic epidemiology groups applied variant-identification pipelines to the same outbreak sequence data. We compared the variants identified by each group in addition to transmission and phylogenetic inferences made with each variant set. To measure the performance of commonly used variant-identification tools, we simulated an outbreak. We compared the performance of three mapping algorithms, five variant callers and two variant filters in recovering true outbreak variants. Finally, we investigated the effect of applying increasingly stringent filters on transmission inferences and phylogenies. We found that variant-calling approaches used by different groups do not recover consistent sets of variants, which can lead to conflicting transmission inferences. Further, performance in recovering true variation varied widely across approaches. While no single variant-identification approach outperforms others in both recovering true genome-wide and outbreak-level variation, variant-identification algorithms calibrated upon real sequence data or that incorporate local reassembly outperform others in recovering true pairwise differences between isolates. The choice of variant filters contributed to extensive differences across pipelines, and applying increasingly stringent filters rapidly eroded the accuracy of transmission inferences and quality of phylogenies reconstructed from outbreak variation. Commonly used approaches to identify *M. tuberculosis* genomic variation have variable performance, particularly when predicting potential transmission links from pairwise genetic distances. Phylogenetic reconstruction may be improved by less stringent variant filtering. Approaches that improve variant identification in repetitive, hypervariable regions, such as long-read assemblies, may improve transmission inference.

## Data Summary

The authors confirm all supporting data, code and protocols have been provided within the article or through supplementary data files. The scripts supporting the conclusions of this article are available in the GitHub repository, https://github.com/ksw9/mtb_variant_identification. The genomic data re-analysed in the Pipeline comparison for epidemic data [1] is publicly available (ENA Study Accession: PRJEB6945). Simulated sequence data and truth VCF files for genome-wide performance benchmarking, in addition to the outbreak phylogeny, multiple sequence alignment and outbreak truth VCF file are available in a digital repository: https://purl.stanford.edu/mr554nj9219.

**Impact Statement**

Pathogens continuously evolve as they spread from person to person. The accumulation of mutations over time can create a valuable epidemiological record that may be used to reconstruct outbreak trajectories and transmission chains. The informativeness of pathogen genomes is contingent on our ability to observe low levels of genetic diversity between closely related pathogens. However, there is no current consensus on how to identify variation within pathogen genomes. We tested whether different approaches in identifying variation in tuberculosis bacterial genomes altered our predictions of potential transmission events. We also measured the performance of commonly used tools in recovering true outbreak variants. We find that variant-identification approaches can substantially alter transmission inferences and that different variant-identification tools vary widely in sensitivity and specificity. Our findings suggest that further work is needed to optimize existing tools for pathogen genomic epidemiology and that long-read sequencing approaches may further enhance the utility of pathogen genomic data.

## Background

The continuous evolution of human pathogens creates a powerful epidemiological record. Patterns of variation within and between populations of pathogens can be used to infer substitution rates, phylogenetic and phylogeographic relationships, such as geographic origins and routes of spatial spread, population size dynamics, and – if pathogen evolution occurs over the same timescale as transmission – transmission patterns [2].

Tuberculosis (TB) kills more people than any other infectious disease and halting transmission of *Mycobacterium tuberculosis* is essential to reducing the global burden of disease. However, in high-incidence settings, it is unknown where and between whom the majority of transmission occurs [3] and therefore where to focus interventions. Molecular epidemiology studies harness genetic and genomic variation to understand patterns of transmission and are premised on the idea that *M. tuberculosis* is constantly evolving as it spreads from person to person. *M. tuberculosis* isolates that share a genotype (RFLP, spoligotype or MIRU-VNTR) [4–6], or which have whole-genome sequences within a given genetic distance [7–10], are considered clustered and potentially epidemiologically linked. Phylogenies inferred from outbreak variation may reveal patterns of relatedness within and between clusters [10–12]. Finally, transmission trees integrate epidemiological and phylogenetic information to capture probable transmission histories, chains of who infected whom [13, 14]. Predicted transmission links have been used to infer the likely location and/or timing [15, 16] of transmission, to identify risk factors for transmission and high-risk populations [17], to distinguish between acquired (primary) and transmitted drug resistance [18] and to declare an outbreak over [19].

Transmission inferences rely on the high-quality measurement of genetic variation from sequence data. However, there is no consensus on how to measure pathogen genomic variation [20]. Molecular epidemiology studies of *M. tuberculosis* often sequence whole genomes directly from bacterial cultures [20, 21]. Sequence data are mapped to a reference genome, variants are identified with respect to the reference and variants are filtered with variant annotation thresholds. The choice of mapping and variant-calling algorithms in addition to variant filters vary widely across studies. Similarly, there is no standard procedure for reference genome selection. While *M. tuberculosis* global diversity falls into seven human-adapted lineages, genomic epidemiology studies frequently use reference genomes from a single lineage, constraining the potential to identify variants that occur outside of the reference-genome backbone. Genomic epidemiology studies of *M. tuberculosis* may additionally apply regional filters, excluding repetitive genes or regions, such as genes in the PE and PPE families [22]. Yet there is no standardized set of genes to exclude.

The *ad hoc* nature of genomic variant calling makes it difficult to interpret pathogen variation within a study and to compare variation across studies. While many pipelines widely used in *M. tuberculosis* molecular epidemiology were designed or validated for antibiotic resistance prediction [22–27], their performance in recovering true pairwise differences and the underlying phylogenetic structure of outbreak genomes, the metrics used for transmission inference, has not been reported. *M. tuberculosis* is slow-growing and only small numbers of substitutions may accumulate over the course of an outbreak [27]. Genomic investigation of *M. tuberculosis* outbreaks is thus uniquely challenging as inferences will be constrained by the sensitivity of tools to detect subtle differences between outbreak strains.

Here, we investigate how different variant-identification approaches may alter *M. tuberculosis* transmission inferences. First, we tested the effect of the variant-calling pipeline on transmission and phylogenetic inferences made from the same sequence data. We collected and compared variant calls from five research groups for the same sequence data from a clonal tuberculosis outbreak in Germany [1]. Second, we measured the performance of variant-calling-tool combinations in recovering genome-wide variants and pairwise differences between outbreak genomes in a simulated TB outbreak for which we knew the underlying genomic truth.

## Results

### Pipeline comparison for epidemic data

**Fig. 1.**
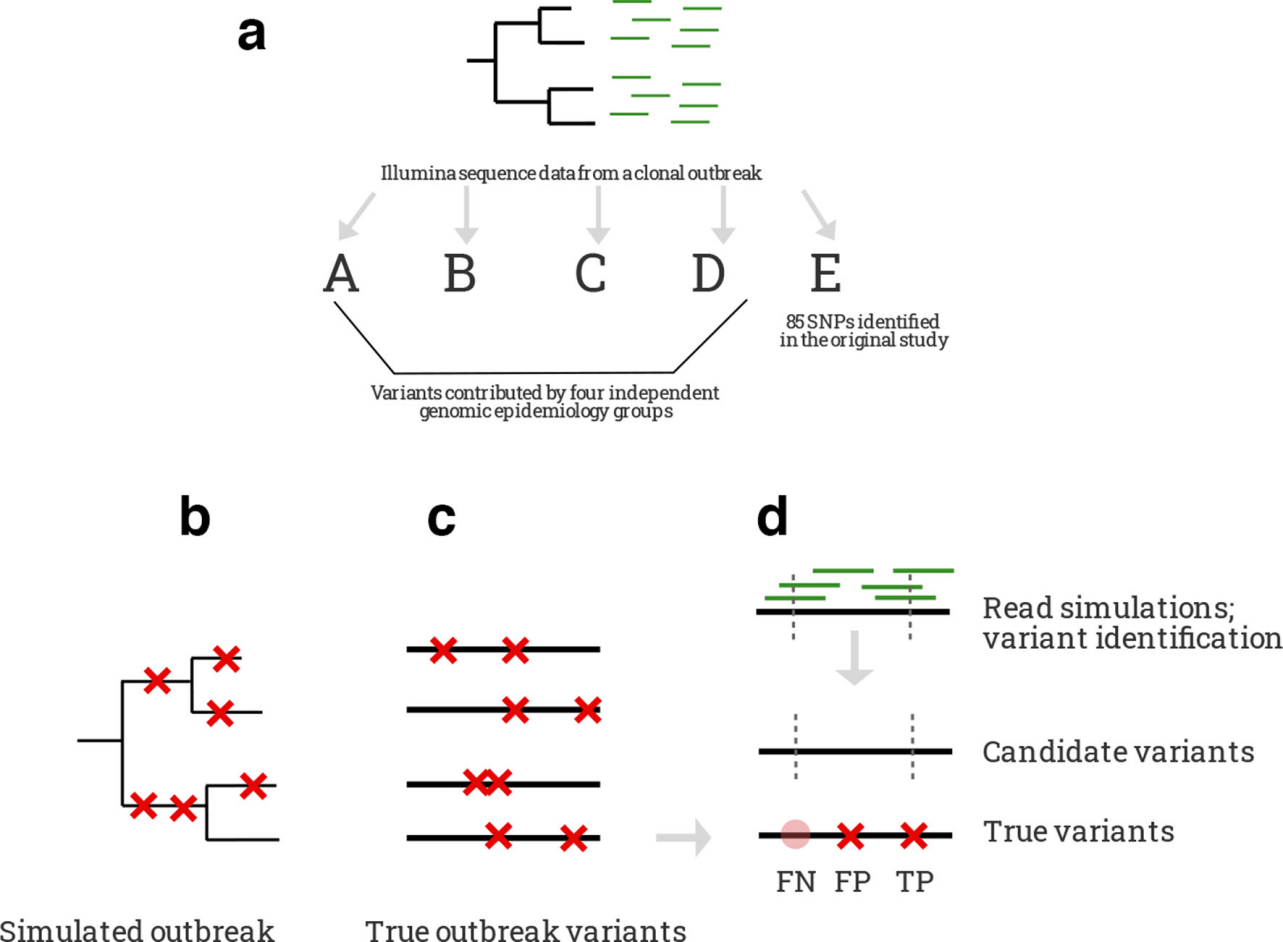
Experimental approach. We investigated the consequences of variant-calling methodological choices on genomic epidemiology inferences with two approaches. First, to test the effect of the variant-identification pipeline on transmission inferences, we conducted a pipeline comparison experiment in which four independent genomic epidemiology groups called variants from the same sequence data generated during a clonal tuberculosis epidemic in Germany (a). We compared variant calls in addition to the transmission and phylogenetic inferences made with each variant set. Second, we measured the performance of variant-calling-tool combinations in recovering genome-wide variants and pairwise differences between outbreak genomes in a simulated TB outbreak for which we knew the underlying genomic truth. We simulated evolution over the course of a model tuberculosis outbreak, generating a phylogeny with known SNP mutations, depicted as red crosses (b). This resulted in a set of closely related full-length outbreak genomes for which we knew the underlying true patterns of genomic variation (c). For each outbreak genome, we simulated Illumina sequence reads (green lines), synthesizing the type of genomic data we might generate in a real outbreak investigation. We mapped reads, called variants, and applied variant filters with several different tool combinations, resulting in 35 sets of candidate variant calls (candidate SNPs depicted as grey dashed lines) for each set of query sequence data (d). We then compared each candidate variant set to the underlying true outbreak variants to evaluate the performance of each tool combination.

To measure the effect of the variant-calling pipeline on transmission inference, four independent genomic epidemiology groups (pipelines A–D) contributed filtered variant calls for previously published sequence data from a clonal *M. tuberculosis* outbreak in Hamburg and Schleswig-Holstein, Germany from 1997–2006 [1] (Fig. 1). The outbreak was identified during routine population-based surveillance and 86 isolates were cultured and fully sequenced on an Illumina platform (ENA Study Accession: PRJEB6945). The original study identified 85 single nucleotide polymorphisms (SNPs) that were validated with Sanger sequencing [1]; we refer to this set of validated SNPs as pipeline E.

All variant-calling pipelines compared here have been applied, formed the basis of, or have been proposed for transmission inference (e.g. pipeline A: [28]; B: [29]; C: [30]; D: [18]) and varied in quality control, choice of reference genome, mapper, caller, variant filters and genomic regions excluded (Table S1, available in the online version of this article).

### Variants identified by different pipelines in the same outbreak data

**Fig. 2.**
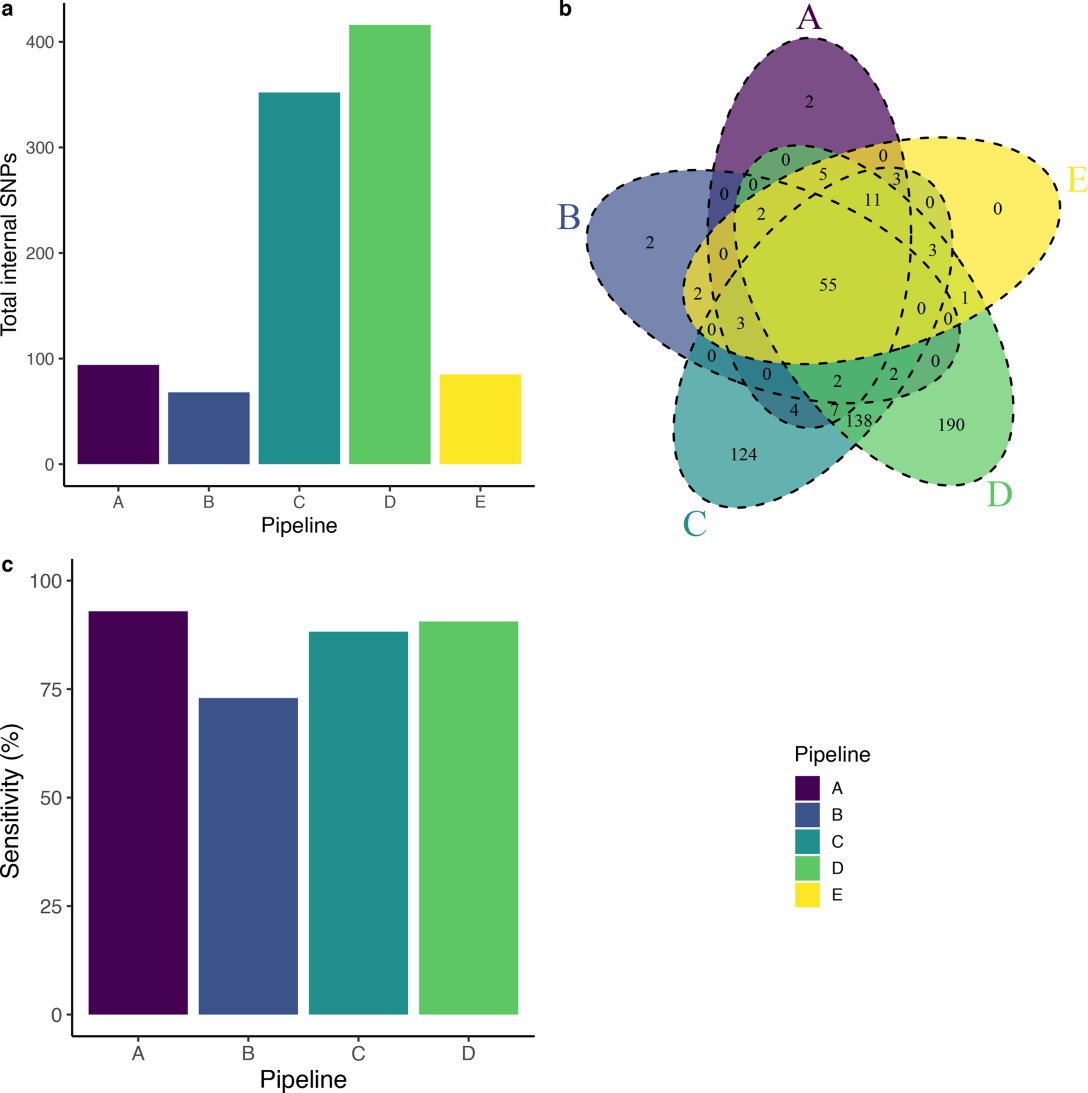
Outbreak variation identified by four pipelines. (a) Total internal SNPs identified by each pipeline (a−d) compared with the 85 SNPs detected in the original study (e), (b) the intersection of SNPs identified by each pipeline, and (c) sensitivity of each pipeline (a−d) in recovering the set of Sanger sequence-verified SNPs from the original study (e).

After filtering, pipelines identified 63 to 416 SNPs between outbreak strains (i.e. internal SNPs) compared to 85 SNPs identified in the initial study (Fig. 2a, Table S2). The five pipelines identified a common set of 55 SNPs (Fig. 2b); however, there was significant discordance in SNPs identified and each pipeline identified 1–190 unique SNPs. Sensitivity in recovering SNPs in the original study ranged from 72.9–92.9 % (Fig. 2c, Table S2). Two variants identified by pipeline B fell in locations on pipeline B’s reference genome (one of the outbreak genomes) that did not correspond to references used by other groups and were unique due to reference choice. Pipeline C excluded 20 % (17/86) of samples that did not meet thresholds for contamination (minimum of 90 % of reads taxonomically classified as *M. tuberculosis* complex) [22]. It is likely that differences in the magnitude of total outbreak variants identified by pipelines C and D result from their treatment of positions of low-coverage or low-quality sequence information, which we discuss further below.

### Transmission inferences across pipelines

Pairwise genetic distances between outbreak sequences, a proxy of the evolutionary distance between genomes, are frequently used to identify *M. tuberculosis* isolates potentially linked by recent transmission [31]. Two isolates separated by a large evolutionary distance are considered unlikely to be the result of recent transmission, while isolates within a threshold genetic distance [7–10] are considered clustered and potentially epidemiologically linked.

While the current consensus is that distance thresholds should be calibrated to the diversity observed within individual studies [20, 31, 32], in practice, previously existing 5- [8] or 12-SNP [7] thresholds are frequently employed to distinguish between ‘clustered’ and ‘non-clustered’ isolates [20].

**Fig. 3.**
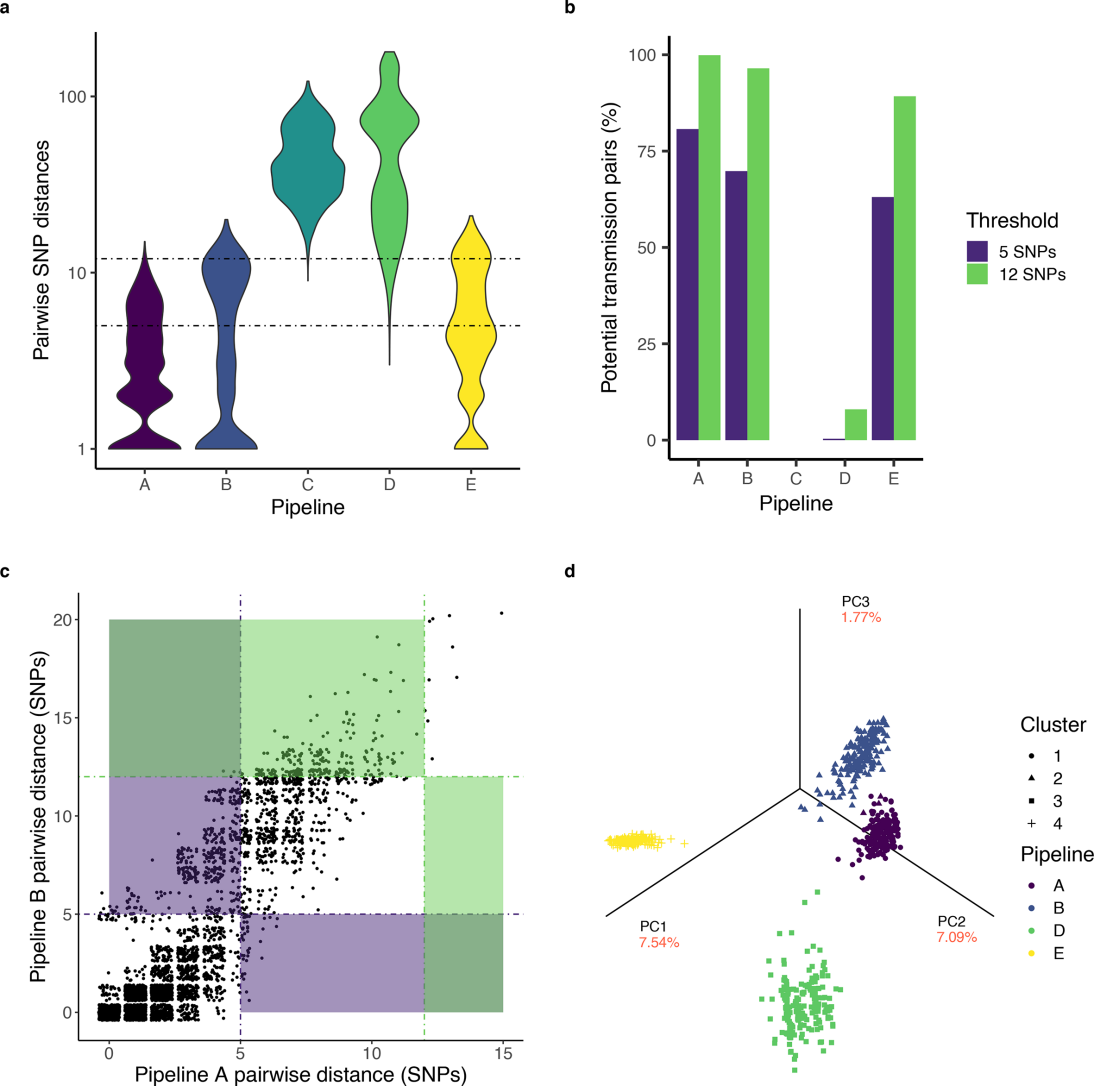
Pairwise SNP distances and phylogenetic trees inferred using different variant-identification pipelines. (a) The distribution of pairwise genetic distances identified by each pipeline on a log-scale. The width of the violin represents the frequency of a given pairwise genetic distance. The dotted lines at 5 and 12 SNPs represent commonly used thresholds for recent transmission. (b) The percentage of sequence pairs with potential transmission links when applying 5 and 12 SNP thresholds for transmission. (c) Pairwise distances between isolates identified by pipelines A and B are closely correlated (Pearson's correlation coefficient, *r*=0.89, *P*<0.001). Each point corresponds to a unique pair of sequences. Dotted lines indicate 5 and 12 SNP distance thresholds and blue and red shading indicates regions in which callers make conflicting transmission inferences after applying a 5 or 12 SNP threshold, respectively. (d) Maximum-likelihood trees inferred from the variation identified by each pipeline largely cluster separately.

The five pipelines identified different distributions of pairwise SNP distances (Fig. 3a), leading to widely different epidemiological interpretations (Figs 3a and b). Median pairwise distances ranged from 1 to 42 SNPs among pipelines (Table S2). Pipelines reported that 0–29.7 % of isolate pairs were identical (0 SNP differences). After applying commonly used transmission thresholds of pairwise distances less than or equal to 5 or 12 SNPs [7, 23, 33], the number of potential transmission links varied dramatically across pipelines (Fig. 3b, Table S2). For example, with variants identified by pipeline A, 80.7 % of sample comparisons fell below a 5-SNP threshold of potential recent transmission whereas with variants identified by pipelines C and D, less than 0.5 % of comparisons did.

Even pipelines that identify similar total numbers of internal SNPs (A and B) and that identify pairwise differences that are closely correlated (Fig. 3c) may still identify different distances between isolate pairs, resulting in conflicting transmission inferences. After applying a 5-SNP threshold for transmission, pipeline A identifies 413 potentially clustered pairs not identified by pipeline B. Conversely, pipeline B identifies 14 potentially clustered pairs not identified by pipeline A. Cumulatively, for the two most similar pipelines, 11.7 % of transmission inferences (427 of 3655 pairwise comparisons) are discordant (Fig. 3c, blue shading). Correlation of measured pairwise differences was lower for all other pipelines (Fig. S1).

Increasingly, transmission inferences are made by incorporating additional epidemiological data along with sequence data. We additionally tested the effect of the variant-identification pipeline on transmission inferences made by *transcluster* [32], a probabilistic approach that integrates sequence alignments, sampling dates and epidemiological priors to predict transmission clusters. We held epidemiologic parameters constant and included sampling dates reported in the original study [1]. Similar to inferences made by applying thresholds to pairwise distances, inferred transmission clusters differed substantially across variant-identification pipelines (Fig. S2). After applying a 5-SNP threshold to transmission clusters, for example, pipelines inferred from 2 (pipelines A and B) to 76 (pipeline D) clusters. We additionally found that adjustment of transmission thresholds does not adequately harmonize transmission inferences across pipelines (Supplementary Text; Fig. S5).

It seems likely that the large number of variants exclusive to pipelines C and D, (Fig. 2), and the apparent similarity of pipeline C and D transmission inferences (Figs S1 and S2) partly reflect pipeline assumptions. Pipelines C and D produced individual sample variant call format (VCF) files that included only variant sites; this precluded distinguishing between reference allele calls and sites with no confident allele call (i.e. at positions of low coverage or quality). To measure pairwise differences between samples, these pipelines assumed that missing sites represented the reference allele, likely generating inflated measures of pairwise differences. An alternative strategy could produce VCFs including all reference-genome positions with information about the depth and quality of reads corresponding to reference and allele calls even at sites with no coverage.

### Phylogenetic inferences across pipelines

We then fit maximum-likelihood phylogenies with alignments of concatenated SNPs identified by each pipeline. We assessed the similarity of bootstrapped trees with Robinson–Foulds (RF) metric, a measure of distance between phylogenetic trees, and used Ward’s method to assign trees into clusters (Fig. 3d). While bootstrap trees do not converge for each pipeline [34], reflecting low levels of measured diversity, the trees inferred by different pipelines are assigned to distinct clusters (Fig. 3d). All trees cluster with their respective pipelines, with the exception of three bootstrap replicate trees inferred from pipeline A variation. Pipeline C, which excludes 20 samples, is not shown because tree distances cannot be computed between trees with different sets of tips.

### Tool performance in a simulated outbreak

For the outbreak described above, as for any outbreak, the true genomic sequence of *M. tuberculosis* isolates is unknown. Performance of pipelines in recovering true outbreak SNPs cannot be measured. Variant-calling pipelines for human genomes are often benchmarked upon diploid human genomic ‘truth sets,’ variants identified and confirmed by several sequencing and bioinformatic pipelines and/or validated by family pedigrees [35, 36]. However, such genomic variant truth sets do not exist for *M. tuberculosis* or other human pathogens.

To evaluate the performance of commonly used variant-calling-tool combinations in recovering genome-wide variants and pairwise differences between outbreak genomes, we simulated sequence data from a set of synthetic, closely related *M. tuberculosis* genomes, for which we knew the underlying true patterns of variation (Fig. 1). We measured accuracy in terms of sensitivity (the probability of true variants are identified) and precision (the probability a variant identified by a caller is indeed a true variant).

We applied commonly used mapping algorithms (BWA, Bowtie 2 and SMALT)[37,38,39] variant callers (Breseq, Pilon, GATK, Samtools and DeepVariant)[40,41,42,43 44] and filters (no filter, a hard quality score filter, QUAL, Pilon-specific filters, and variant quality-score recalibration, VQSR) to simulate data and measure performance in recovering true SNP variants (Methods).

### Performance in recovering *M. tuberculosis* SNPs across tool combinations

To measure performance of variant-calling tools in recovering genome-wide *M. tuberculosis* variants, we generated 20 sets of Illumina short-read data *in silico* from the *M. tuberculosis* strain CDC1551 query genome and evaluated the performance of nine variant-calling-tool combinations in recovering the 1501 SNPs identified by pairwise alignment of the query and the frequently used *M. tuberculosis* strain H37Rv reference genome (Methods).

**Fig. 4.**
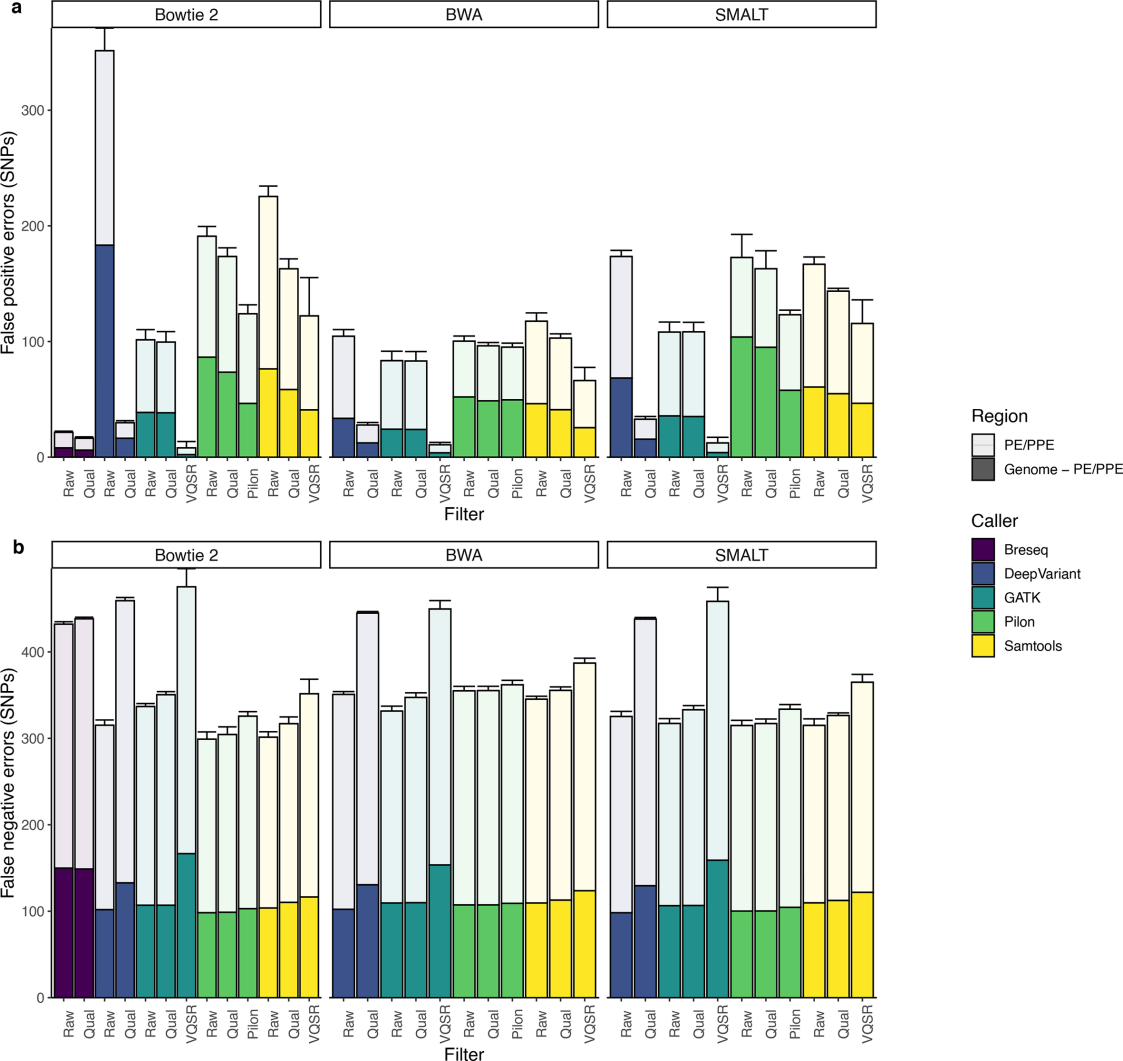
Errors in *M. tuberculosis* SNPs identified by different tool combinations. Mean and standard deviation of false positive (a) and false negative (b) errors identified by variant-identification tool combinations. Breseq is a complete computational pipeline that includes mapping with Bowtie 2. Bar colour indicates mapper and shading indicates genomic region. Panels have different y-axes.

Performance in recovering true genome-wide *M. tuberculosis* SNPs varies widely across tool combinations (Fig. 4) using strain H37Rv as the mapping reference. Prior to filtering, variation in precision exceeds that of sensitivity; maximum precision is 98.0 % (Bowtie 2/Breseq) while maximum sensitivity is 80.1 % (Bowtie 2/Pilon) (Table S3). The number of false positive (FP) errors varies from 21.4 (Bowtie 2/Breseq) to 351 (Bowtie 2/DeepVariant) before filtering.

We examined the genomic location of errors and tested if standard filters could reduce FP errors. Variant-calling performance varies across the genome and is worse in the 168 repetitive PE/PPE genes (Fig. 4), which are often excluded from *M. tuberculosis* molecular epidemiology studies [45]. Before filtering, 39.8–71.0 % (identified by SMALT/Pilon and BWA/GATK, respectively) of FPs occur in PE/PPE genes, which comprise 6.37 % of the genome (Fig. 4a). False negative (FN) errors are also disproportionately located in the PE/PPE genes (Fig. 4b). Before filtering, 65.2–70.8 % (identified by (SMALT/Samtools and BWA/DeepVariant, respectively) of FNs occur in PE/PPE genes.

Filtering by excluding the PE/PPE genes or by filtering by quality score or VQSR reduces but does not eliminate FP errors, while increasing FN errors. FP errors are minimized by Bowtie 2/GATK with VQSR and excluding the PE/PPE genes (mean FP 2.2 SNPs, mean FN 167 SNPs). Even when PE/PPE genes are included, GATK/VQSR tool combinations identify fewer FP errors than all other tool combinations (Fig. 4). FN errors are minimized by SMALT/DeepVariant, excluding the PE/PPE genes (mean FP 68.4 SNPs, mean FN 98.2 SNPs). Further, filters contribute to extensive variation across tool combinations. For example, applying a VQSR filter as compared to a quality filter to calls identified by BWA/GATK can reduce FP errors from 83 to 10 (when still including PE/PPE genes) (Fig. 4).

We additionally examined the source of FN errors for Pilon/Bowtie 2, the tool combination with the highest sensitivity (lowest FN errors) to determine if FNs could be attributed to filtering or incorrect reference allele calls. Of the mean 327 total FN errors (Table S3) an average of 50.2 positions were called as heterozygous sites by Pilon. These sites were marked as ‘Ambiguous’ by Pilon and filtered. However, the majority of FN sites could not be explained by ambiguous calls. A mean of 206.0 sites along the genome were marked as low coverage and 99.1 sites were marked as deletions by Pilon and were additionally filtered. Many of these occurred in the repetitive PE/PPE genes. Finally, many FN errors are adjacent to sites called as indels. This suggests that further optimization of filters to specific tool combinations, in addition to post-processing steps such as the normalization of variant calls, could improve performance beyond that achieved using the default parameters. Our intent here is not to optimize the parameters for each caller; but rather to test whether variants identified and performance vary across tool combinations.

All tool combinations are characterized by a trade-off between sensitivity and precision visible in the inverse relationship between FP and FN errors. However, no tool combination consistently outperforms other tool combinations in minimizing both types of errors (Fig. 4), indicating that an optimal approach may depend on the relative costs of different error types for specific applications. No combination of mapper, variant caller and filter were able to achieve >99.9 % precision and sensitivity reported for human genomes and which won the PrecisionFDA Truth Challenge, a competition in small variant identification from short-read genomic sequence data [44].

### Effect of reference choice on performance in recovering *M. tuberculosis* SNPs

To assess how the choice of reference genome affects variant-calling performance, we mapped one sequence set to 13 different reference genomes spanning global *M. tuberculosis* diversity and ranging from 1376 (lineage 4, strain F11) to 3396 (lineage 2, strain Beijing_NITR203) SNPs distant from the strain CDC1551 query genome (lineage 4, Table S4). In Poisson-generalized linear models, log-transformed distance to the reference genome, mapper and caller are significant predictors of both FP and FN errors, prior to filtering.

Both FP and FN errors increase with increasing log-transformed distance between the query and reference genomes, when controlling for pipeline (Fig. S3). Errors vary widely between reference genomes, possibly reflecting individual genomes’ repetitive content, extent of synteny with the query genome, or reference assembly quality.

### Performance in recovering pairwise differences across tool combinations

Studies of the genomic correlates of antibiotic resistance or virulence seek to identify variants in a single genome with respect to a reference genome. In contrast, variant calling for transmission inference seeks to measure small amounts of variation between multiple closely related outbreak genomes. Identifying variants between query genomes and a known reference genome is intermediate to the true goal: identifying variants between the outbreak genomes. If errors with respect to the reference genome are consistent within a single pipeline, then inference about relatedness between outbreak samples should not be affected.

We measured the performance of tool combinations in identifying pairwise differences between closely related sequences within a model 5 year TB outbreak (Methods). We simulated evolution of *M. tuberculosis* from a common ancestral genome (strain CDC1551) over the outbreak phylogeny (Figs S4 and S6), resulting in a total of 145 outbreak SNPs with respect to the strain H37Rv reference, and generated sequence data *in silico* from the 44 outbreak sequences. True pairwise differences between outbreak genomes ranged from 0 to 27 SNPs and mean pairwise distance between isolates was 13.2 SNPs with respect to the reference genome.

**Fig. 5.**
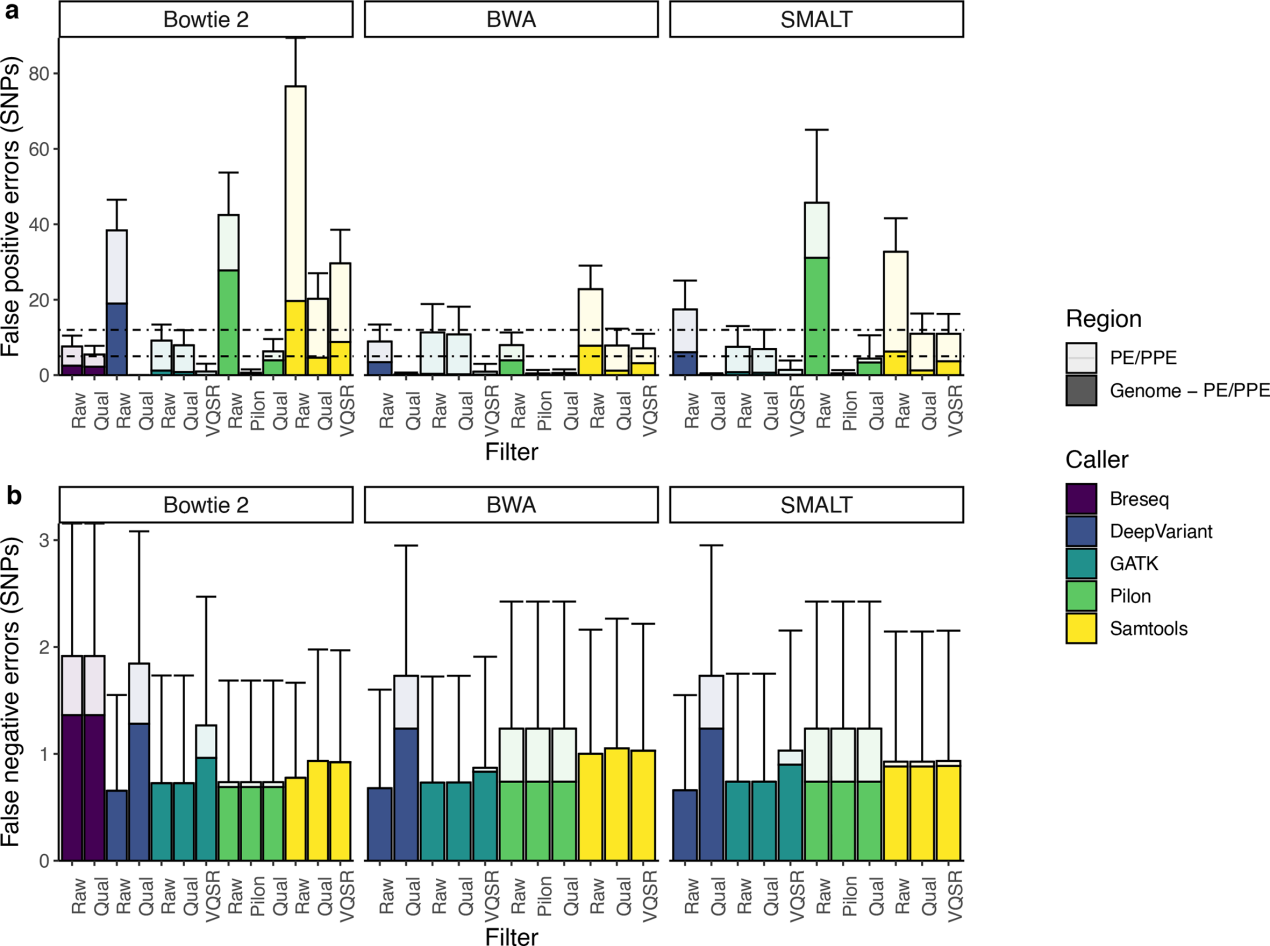
Errors in pairwise differences identified by different tool combinations. Mean and standard deviation of false positive (a) and false negative (b) SNP differences between outbreak sequences identified by variant-identification tool combinations. Breseq is a complete computational pipeline that includes mapping with Bowtie 2. Bar colour indicates mapper and shading indicates genomic region. Dotted lines in (a) indicate 5 and 12 SNP distance thresholds, commonly used for inferring recent transmission. Panels have different y-axes.

Performance in recovering true pairwise differences between outbreak strains varied across tool combinations using strain H37Rv as the mapping reference. Prior to filtering, mean sensitivity ranges from 85.9 % (Bowtie 2/Breseq) to 95.0 % (Bowtie 2/DeepVariant) and mean precision ranges from 14.7 % (Bowtie 2/Samtools) to 64.5 % (SMALT/GATK) (Table S5). As seen for genome-wide performance, performance in identifying pairwise differences is worse in the 168 repetitive PE/PPE genes compared to the rest of the genome. Before filtering, 31.9 % (SMALT/Pilon) – 97.0 % (BWA/GATK) of FPs occur in PE/PPE genes (Fig. 5).

We then tested whether filters could improve performance in recovering pairwise differences. Quality score or VQSR filters reduce but do not eliminate FP pairwise errors (Fig. 5, Table S5). Several tool combinations result in mean FP pairwise errors above 5 SNPs if PE/PPE genes are not excluded (i.e. approaches with Samtools; GATK/QUAL; Breseq/QUAL; Bowtie 2/Pilon/Qual; BWA/Pilon/Pilon). If a pairwise difference threshold of 5 SNPs was applied, the effect of variant-calling errors alone would exclude the possibility of recent transmission. Even after filtering, the range of mean FP errors is more than 25 times that of FN errors across tool combinations (Fig. 5).

Because 23 of the 145 outbreak SNPs occur within PE/PPE genes, approaches which exclude PE/PPE genes have a maximum total sensitivity of only 84.1 % (122/145) of the total outbreak variation. Tool combinations including GATK/VQSR, DeepVariant/QUAL, and Bowtie 2 or SMALT with Pilon and Pilon-specific filter or SMALT/Pilon/Qual allow PE/PPE gene variation to be retained while keeping mean FP errors below 5 SNPs. Among tool combinations that include PE/PPE gene variation and with mean FP errors below 5 SNPs, maximum sensitivity was 94.1 % (Bowtie 2/Pilon/Pilon) and maximum precision was >99.9 % (Bowtie 2/DeepVariant/QUAL). We found that pairwise errors are often repeated and cluster within repetitive genomic regions (Supplementary Text).

### Effect of variant filtering on transmission inferences

Variant filters vary widely between studies and can contribute more to variation between tool combinations than either mapping or variant calling (Figs 3 and 4). However, filters are frequently not justified empirically, and the effect of filtering on transmission and phylogenetic inference is unknown. To test the effect of variant filtering on downstream inferences, we applied a series of increasingly stringent quality-score filters to variant calls identified by a single tool combination, BWA/GATK.

**Fig. 6.**
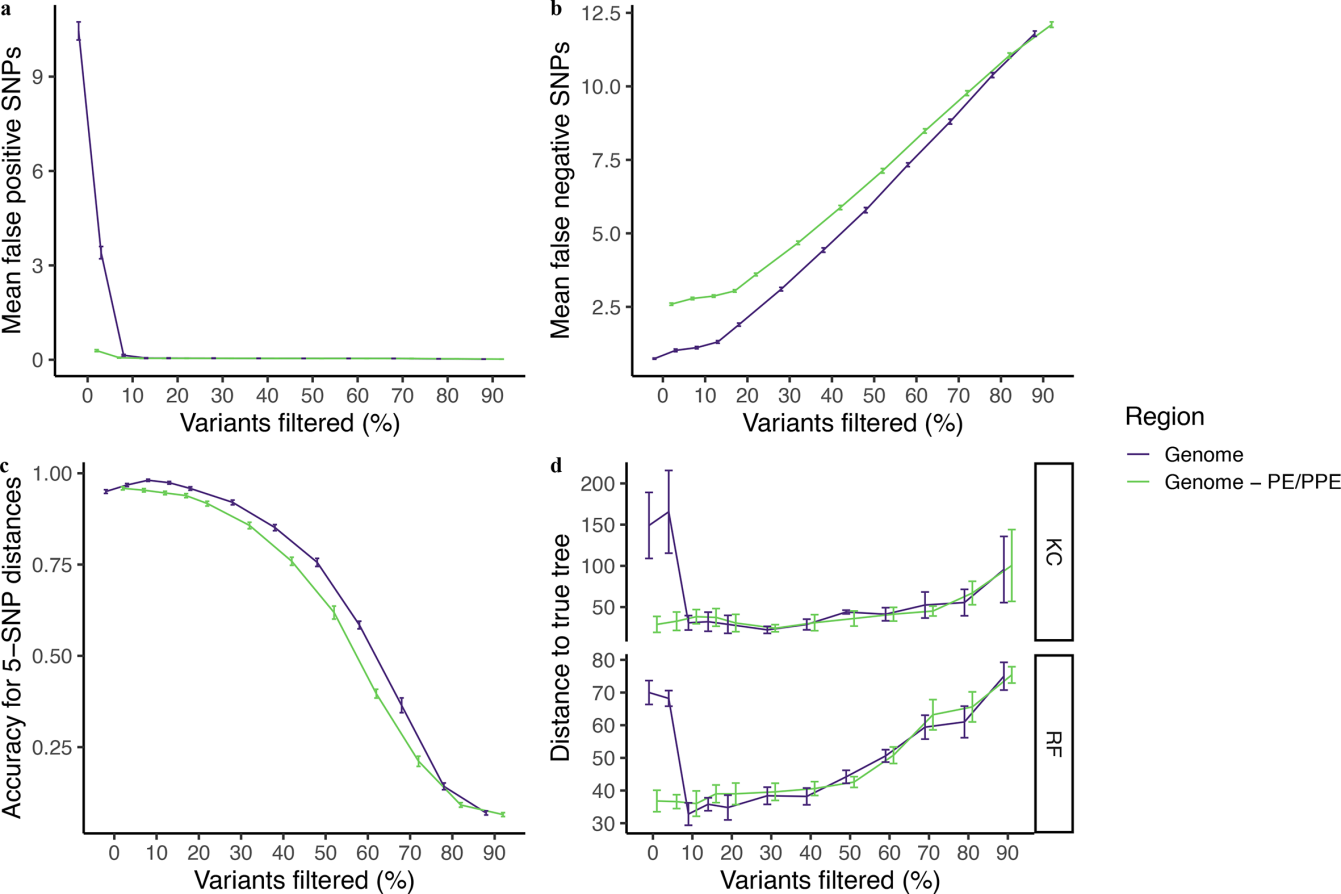
Effect of increased filtering on transmission inference and phylogenetic reconstruction. (a) False positive and (b) false negative pairwise errors identified in the simulated TB outbreak sequences with BWA/GATK and increasingly strict variant filtering. (c) Accuracy in distinguishing pairs falling above or below a 5-SNP threshold. (d) Distance of phylogenies inferred with increasingly filtered variants to the true outbreak phylogeny measured with the KC [65] and RF [55] metrics. Genomic region is indicated by colour and points corresponding to each genomic region are staggered along the x-axis to improve clarity. Points represent mean pairwise errors for each filtering level and error bars indicate the partially pooled errors across ten replicate sequence sets.

As expected, applying increasingly strict variant quality-score filters reduces observed pairwise differences between outbreak samples, resulting in a trade-off between FP and FN errors (Fig. 6a, b). Mean genome-wide FP pairwise errors are 10.5 SNPs before quality filtering and 0.29 SNPs after excluding the PE/PPE genes. Mean genome-wide FP errors fall rapidly to 0.14 after excluding variants in the lowest quality decile and 0.046 SNPs after excluding PE/PPE genes. Mean genome-wide FN errors are 0.74 before filtering and increase rapidly after excluding the lowest two deciles of variants. FN errors are consistently higher in variant sets excluding PE/PPE genes, reflecting the fact that 15.9 % (23/145) of true variants occur in these genes.

Before quality filtering, 95.0 % of isolate pairs were correctly assigned as falling above or below a 5-SNP threshold when considering genome-wide variants; 95.9 % pairs were correctly assigned after exclusion of the PE/PPE genes (Fig. 6c). Accuracy in distinguishing pairs falling above or below a 5-SNP threshold improves slightly after excluding variants in the lowest quality decile to a maximum of 98.1 % for genome-wide variants and 94.6 % after excluding PE/PPE genes, after which accuracy rapidly declines. Excluding the PE/PPE genes generally results in lower accuracy in identifying isolate pairs falling under a 5-SNP threshold (Fig. 6c).

Distances of reconstructed trees to the true underlying phylogeny fall rapidly after initial filtering and then steadily increase with more stringent quality score filters, resulting in a U-shaped relationship between quality-score filter and distance to the true tree, measured by KC distance, and a hockey-stick-shaped relationship for RF distance (Fig. 6d). When no quality filtering is applied, the inclusion of variants within PE/PPE genes results in large distances of inferred phylogenies to the true tree (149.0, KC distance and 70.0, RF distance). Mean KC tree distances fall to a minimum of 22.4 after filtering 30 % of variants, when genome-wide variants are included. Mean RF distances fall to a minimum of 35.8 after filtering of 10 % of variants, when genome-wide variants are included. These observations suggest that some filtering is necessary to remove the lowest quality variants, either by exclusion of problematic regions or by exclusion of the lowest quality variants, but additional filtering may rapidly erode the quality of inferred phylogenies.

## Discussion

As shown in the results (pipeline comparison for epidemic data), methodological differences between different *M. tuberculosis* molecular epidemiology groups can lead to differing epidemiological conclusions made from the same sequence data. While a recent study found that four European variant-identification pipelines were largely concordant in their ability to rule out potential transmission links between *M. tuberculosis* isolates [46], the pipelines in this earlier study applied similar genomic filters and three of the four pipelines compared used Samtools. Further, while the earlier study compares the number of genomically clustered isolates identified by different pipelines, here, we compare the pairwise distances identified by different pipelines.

Our findings suggest that results from genomic epidemiology studies need to be interpreted in the context of study methodology. The lack of reproducibility among variant-identification pipelines affects all downstream analyses, particularly as variant uncertainty is not often reported or incorporated into other analyses. Estimates of *M. tuberculosis* substitution rate, for example, are similarly contingent on variant-calling pipeline. Stringent filtering or the exclusion of variants within the PE/PPE genes will likely decrease the observed molecular clock rate of *M. tuberculosis *. We note several limitations to our study (Box 1), including that we focus only on the impact of variant-identification methods on transmission inferences and not other potential downstream inferences.

Box 1. **Study limitations**

- We measured performance on simulated sequence data that includes errors associated with Illumina sequencing [57], but which likely does not capture the full spectrum of sequence errors introduced in epidemiological studies.
- We do not examine indels and other structural variants, which data presented by [66] demonstrate can be phylogenetically informative.
- We do not measure performance of pipelines in identifying within-host variation, which is clinically and epidemiologically important [67, 68] and can provide additional information for transmission inference [69, 70]
- We did not investigate the performance of variant-calling tools in recovering variants associated with antibiotic resistance or other phenotypes.
- We did not measure the effect of recombination within an outbreak or with respect to a reference genome on the ability of tools to recover outbreak SNP variation.
- Performance measures of variant-identification tools depend on the accuracy of the truth set, generated by pairwise alignment of query and reference genomes. As described in Methods, we found that pairwise alignment had sensitivity >98 % and precision >95 % when recovering introduced SNP variants between closely related genomes. Because of this uncertainty in the truthset, some of the candidate genome-wide variants in the ‘Tool performance in a simulated outbreak,’ may be misclassified.

### Trained variant-identification tools

Sequencing technologies and variant-calling algorithms are rapidly changing, and our aim was not to identify a single best pipeline, but instead to characterize the reproducibility and accuracy of variant-identification tools when applied with default settings. We found that performance varies widely across approaches and that no single tool combination out-performs all others. The good performance of DeepVariant and Pilon in recovering outbreak variation likely reflects calibration upon labelled sequence data, through the training of a neural network (DeepVariant) or fitting of Gaussian mixture models to variant annotations (GATK/VQSR), or for Pilon, the use of read-pair information to improve local assembly, particularly in repetitive regions [41].

### Interpreting population-level variation

A significant source of differences among pipelines in the results (pipeline comparison for epidemic data) could be attributed to the interpretation of variants following variant calling. This highlights the need to standardize reporting of variants and distinguish between missing sites and reference allele calls.

### Variant filtering

We found that subtle differences between outbreak genomes can be readily overwhelmed by bioinformatic errors. However, appropriate filtering can greatly reduce both false-positive and false-negative errors while, in some cases, retaining variation in PE/PPE genes. Filters contribute to extensive variation across tool combinations. Further, our results demonstrate that tools and filters interact.

Many genomic epidemiology studies employ some type of hard filtering, whether based on annotation or genomic region. Transmission inferences based on pairwise differences as well as phylogenies are sensitive to variant-filtering strategy and optimal filters may depend on specific downstream application. While minimal filtering improves the accuracy of transmission linkages predicted by pairwise differences and tree reconstruction, extensive filtering results in poorer accuracy of predicted transmission linkages and phylogenies that are increasingly distant from the underlying true phylogeny. After limited quality filtering, including the PE/PPE genes does not negatively affect transmission or phylogenetic inferences. The PE/PPE genes are the most variant dense regions of the *M. tuberculosis* genome and are known antigens and virulence determinants [45]. Routine exclusion of these genes reduces the information potential of *M. tuberculosis* genomes and limits our ability to study the functional consequences of *M. tuberculosis* variation. Our findings suggest that, if appropriate filters are applied, it may be possible to retain variation in many of the PE/PPE genes, frequently excluded from genomic epidemiology studies.

### Reference genome choice

As expected, variant errors increased with increased distances between the reference and query genomes. This contrasts with a previous study that found the choice of reference genome did not affect *M. tuberculosis* epidemiological inferences [47]. Our study differs from the previous study in that we used simulated genomic data for which underlying true variation is known to measure performance in identifying variants in individual genomes. The earlier study measured how reference choice affects performance in classifying isolate pairs as linked or unlinked using the strain CDC1551 reference genome as truth.

*M. tuberculosis* genomic epidemiology studies routinely use strain H37Rv or strain CDC1551 reference genomes, both of which belong to lineage 4. Studies investigating variation in other lineages will particularly benefit from using local reference genomes, either a full-length genome from the outbreak being studied or another closely related genome. Gene content differs between *M. tuberculosis* lineages [20, 48], constraining sensitivity in a reference-based genome approach. Any variation within regions inserted in the query genomes relative to the reference will be missed even by a perfectly sensitive variant caller.

### Pathogen genomic epidemiology needs

The issues we identify here generalize to other pathogens, particularly those with slow relative rates of substitution compared with time course of transmission. Our results suggest that pathogen genomic epidemiology, for *M. tuberculosis* and other species, will benefit from the further optimization of genomic resources and methods for bacterial genomes and the use of long-read sequencing data.

First, pathogen genomic truth sets of experimental (not simulated) sequence data accompanied by validated variants would enable training of machine-learning approaches upon labelled pathogen variant data and would serve as a gold standard for performance benchmarking of variant-calling approaches. Secondly, further work is needed to optimize variant callers for pathogens and for particular applications (i.e. prediction of antibiotic resistance versus transmission inference). Further, variant callers could output quality scores for reference allele calls in addition to alternative allele calls, enabling comparisons between all sites (as done by GATK and Pilon). This allows filters to be applied to both reference allele calls and alternate allele calls (i.e. reference allele calls might have poor coverage and/or quality just as alternate alleles might). Third, we find that filtering on site and sample-specific annotations allows all available information about variants across samples to be retained (i.e. many variant-calling programs ‘merge’ sample-specific annotations into a maximum or mean annotation for a site and information about a low-quality call for a single sample may be lost). Fourth, the power of long-read sequence data could improve accuracy of transmission inferences. Finally, variant uncertainty represents an important and unreported source of potential error in genomic epidemiology studies. How to incorporate uncertainty in underlying measures of genomic variants or sequences in phylogenetic inference remains an open and important question.

Other groups have identified methods to reduce additional sources of error in genomic epidemiology studies. For example, taxonomic filtering can importantly exclude reads from contaminating microbial species [49]. Additionally, other work has found that calling variants for samples independently rather than jointly may improve sensitivity for detecting low-frequency microbial variants [50].

### Conclusions

While many applications of *M. tuberculosis* whole-genome sequencing for transmission inference use hard filters to minimize false-positive SNPs [20] and then apply pairwise SNP distance thresholds to infer potential transmission linkages [20, 51], here we show that (a) such approaches do not recover consistent sets of SNPs; (b) pairwise distance thresholds are not robust to differences between pipelines; and (c) strict filtering does not always improve transmission inferences made using pairwise differences or phylogenies.

We conclude that measurements of genetic distance and phylogenetic structure are dependent on variant-calling approach. More generally, we find that pathogen genomic epidemiology studies will benefit from genomic resources and tools designed for haploid genomes.

## Methods

### Pipelines

Groups submitted filtered variant calls as single-sample or multi-sample VCFs in addition to a multiple sequence alignment of concatenated SNPs. We used LiftoverVcf (http://broadinstitute.github.io/picard/) to convert variant coordinates for pipelines A and B to coordinates on the strain H37Rv reference genome so that variant positions could be compared.

Pipeline C made diploid calls and did not provide a multiple sequence alignment. To create a multiple-sequence alignment of consensus sequences, we converted diploid calls to haploid by setting homozygous genotypes (0/0 or 1/1) to the corresponding haploid genotype (0 or 1) and heterozygous genotypes to the genotype with greater allele depth. We used bcftools [43] to generate consensus sequences, setting genotypes that were absent in single-sample VCFs to the reference allele, as specified by the authors.

Pipeline A additionally included diploid calls, however, also provided a FASTA file used for pairwise differences and phylogenies in addition to a list of variant sites internal to the outbreak. We used the list of variant sites internal to the outbreak to compare variant sites with other pipelines.

### Sensitivity

The original study confirmed ﻿85 internal outbreak SNPs with Sanger sequencing. While the underlying true number of outbreak SNPs is unknown, Sanger sequencing provides a high degree of confidence in this subset of SNPs. We therefore report pipelines’ ‘partial sensitivity’ in recovering these high-confidence, previously identified SNPs. Specificity could not be measured.

### Transmission cluster inference

We inferred transmission clusters based on both SNP distances (SNP clusters) and with the R package *transcluster* v0.1.0 (transmission clusters) [32], which integrates sequence alignments, sampling dates and epidemiological priors to predict transmission clusters. We held epidemiological parameters constant, specifying a clock rate of 1.5 substitutions/genome/year (within the range of recently reported substitution rates [52]) and a transmission rate of 2.3 transmissions/year (within the range of potential transmission rates) and set both SNP-clustering and transmission cluster thresholds to 5 and 12 SNPs.

### Phylogenetic inference

We calculated raw pairwise differences between isolates with the R package ape v.5.2 (model = ‘N’) [53]. We constructed maximum-likelihood phylogenies with RAxML-ng [54] with a GTR substitution model. We used a Stamatakis ascertainment bias correction to correct for invariant sites and specified nucleotide stationary frequencies present in the strain H37Rv genome. We measured phylogenetic distances with the RF metric between a random selection of 200 bootstrap replicate trees derived from SNPs from each pipeline [55]. We reduced the dimensionality of tree distances with principal components analysis using the R package treespace [56]. To summarize the multi-dimensional distances between trees inferred with variants from each pipeline, we performed hierarchical clustering of trees using Ward’s method also in treespace [56].

### Tool performance in a simulated outbreak

We simulated a tuberculosis outbreak and generated sequence data *in silico* (Figs S4 and S6). We applied commonly used mapping and variant-calling algorithms to simulated data and measured the performance of these variant-calling-tool combinations in recovering both true *M. tuberculosis* genomic variants and true pairwise differences between closely related *M. tuberculosis* sequences.

### Simulated sequence data

We generated 20 independent Illumina readsets (2×151 bp) from the *M. tuberculosis* strain CDC1551 genome in silico, with the next-generation sequence-read simulator ART v. 2.5.8 [57], which simulates reads from a given genome with read lengths and error profiles from commonly used sequence platforms. We simulated reads using a built-in quality profile for a HiSeqX v2.5 TruSeq sequencing machine. Before simulations, we set ambiguous sites in the strain CDC1551 genome to N. We simulated reads with a mean of 100X coverage, with a mean and standard deviation fragment length of 650 and 150 bp, respectively (consistent with Illumina recommended insert sizes of 350 bp (https://support.illumina.com/sequencing/sequencing_instruments/hiseq-x/questions.html; standard deviation from empirical data).

Measuring performance requires a truth VCF of true variant sites in the query genome with respect to a given reference genome (Fig. 1). To generate truth VCFs for the strain CDC1551 query genome with respect to 13 *M *. *tuberculosis* reference genomes (Table S4), we pairwise aligned the query genome (strain CDC1551) to each reference genome separately with MUMmer [58] (*nucmer maxmatch -c 1500*), generating 13 pairwise alignments. We identified SNP variants from the pairwise alignments using MUMmer *show-snps*, excluding SNPs with ambiguous mapping and indels (*show-snps -CIr*).

Pairwise alignment does not perfectly recover all SNP variants between genomes. To test the accuracy of the pairwise alignment algorithm in identifying true SNP differences between the query and reference genomes, we created a query genome, which we refer to as H37Rv* by introducing a ‘test set’ of known SNP and indel variants into the strain H37Rv backbone with GATK’s FastaAlternativeReferenceMaker. To select a test set of SNPs that were representative of standing biological variation in *M. tuberculosis *, we used the variants identified by the tool combination BWA/GATK/QUAL >40, in strain CDC1551 with respect to strain H37Rv, including 1232 SNPs and 376 indels. We then pairwise aligned the mutated genome, H37Rv* (mutated at the test set positions), to the strain H37Rv reference genome with the MUMmer method described above and measured sensitivity and precision of the pairwise aligner in recovering the 1232 introduced SNPs in the test set. Sensitivity was 98.8 % (1217/1232) and precision was 95.1 %(1217/1280). The errors in our method of defining a ‘truth VCF’ therefore means that there will be some measurable misclassification of candidate genome-wide variants identified by the different tool combinations.

### Mapping and variant calling

We mapped simulated reads with three read mappers, bwa[37], Bowtie 2 [38], and SMALT [39], using default settings. We mapped reads to strain H37Rv in addition to 13 other reference genomes spanning described *M. tuberculosis* diversity (Table S4). We called variants with five variant callers using default parameters unless otherwise specified. For GATK, Samtools and Pilon, we set ploidy to 1. The Breseq computational pipeline[40] includes mapping with Bowtie 2 and variant calling; therefore, Breseq is calls are not made in combination with other mappers. We called variants for each sample independently rather than jointly calling genotypes because joint variant-calling approaches are designed for human cohort studies and were found to be less sensitive in detecting singleton and low-frequency variants in a previous study [50]. We excluded all sites flagged by Pilon as ambiguous (i.e. mixed allele calls) or deletions.

We additionally called variants for each sample independently with DeepVariant v.0.7.0, a convolutional neural network trained upon human genomic truth sets to identify variants in short-read sequence data [44]. Specifically, DeepVariant v.0.7.0 was trained upon labelled genotypes from a total of 16 sets of human genomic data, including ten PCR-free sequence replicates of HG001, two PCR-free replicates of HG005 PCR-free and four PCR replicates of HG001. The genomic ‘truth’, which the model is trained on, includes variants that have been identified by several pipelines and occur within ‘high confidence’ regions of the human genome [59]. The model was frozen after training and then can be applied to unseen genomic data in the form of aligned reads (BAM files). DeepVariant does not have an option to infer haploid genotypes; therefore, we assigned homozygous genotype predictions to the corresponding haploid call (i.e. assigning 0/0 to 0 and 1/1 to 1). For heterozygous calls, we used allele depth to assign genotype as the allele with greater coverage. If two alleles at a heterozygous site had equal depth, we randomly selected a haploid genotype. We set DeepVariant SNPs filtered as ‘RefCall’ to missing. For all callers, we output all-sites VCF files (i.e. both variant and invariant sites) in order to distinguish between reference allele calls and missing or ‘no-call.’ Software tools are listed in Table S6.

### Variant filtering

We excluded indels and applied two independent filters to SNP variant calls to samples individually: a single hard variant quality-score filter, QUAL <40 and VQSR (variant quality-score recalibration) [42]. VQSR fits Gaussian mixture models to annotations characterizing a truth set of high-quality variants and then applies this model to all candidate variants to recalibrate variant quality scores. Because a high-quality truth set does not exist for *M. tuberculosis *, we defined our truth set internally, including all candidate SNPs with a QUAL score greater than the mean QUAL score for a given set of variants. We set a phred-scaled prior likelihood of 15 and used the annotations DP, QD, MQRankSum, ReadPosRankSum, FS, SOR and MQ in the model. We set the recalibrated variant quality-score (VQSLOD) threshold so that our caller would have 99 % sensitivity for recovering variants within our truth set. We did not apply VQSR to DeepVariant calls to avoid overfitting. By default, Pilon flags low-coverage variants (default minimum depth is 10 % mean coverage or 5X, whichever is greater). We additionally filtered these low-coverage variants from Pilon calls only, referring to this as the ‘Pilon’ filter for Figs 3 and 4.

### Performance benchmarking

We used hap.py [60], software widely used to measure performance of variant-calling pipelines upon human genomic variation [44], to assess the performance of each pipeline.

### Outbreak simulations

We simulated a short, relatively densely sampled 5 year TB outbreak with TransPhylo [13, 61] (Fig. S4). We set the basic reproduction number, *R*_0_, to be 3 and the generation time, the time between subsequent infections, and sampling time, time between infection and diagnosis, as Gamma distributed, with shape of 10 and scale of 0.1, corresponding to a mean of 1 year. We set the product of the within-host population size and generation time (*N*_e_ *g*) to 100/365 and the probability of observing cases, π, to 0.25.

TransPhylo simulates transmission trees, graphs of who infected whom and when in an outbreak. We extracted the underlying phylogeny from the simulated transmission tree using a substitution rate estimate of 2 substitutions/genome/year [52]. We chose a rate on the higher end of published clock rates for *M. tuberculosis* to ensure that we would obtain sufficient numbers of ‘true’ simulated SNPs on which to test both pipelines and downstream inference.

To generate a set whole-genome sequences related by the simulated genealogy, we then simulated evolution along the simulated phylogeny with Pyvolve [62]. Pyvolve takes a phylogeny, a root sequence, and a nucleotide substitution model and simulates evolution along the branches of a phylogeny. We simulated nucleotide evolution from the strain CDC1551 reference genome with an F81 model of nucleotide evolution [63] with empirically derived nucleotide frequencies. We used snp-sites [64] to generate a VCF file of variant sites in the tip genomes. Pyvolve introduces variants randomly along the root sequence; to simulate variation at sites known to be polymorphic in *M. tuberculosis *, we replaced the sites simulated with Pyvolve with randomly selected sites that varied between strains CDC1551 and H37Rv, allowing us to preserve the simulated phylogenetic structure while including variants that are segregating in natural *M. tuberculosis* populations. The clustering of true outbreak variants reflects that they were drawn from a set of sites polymorphic between the strains H37Rv and CDC1551 reference genomes. We applied this set of 147 SNPs to the strain CDC1551 reference genome, generating 44 simulated outbreak sequences. We then used LiftOver to generate a ‘truth’ outbreak VCF with respect to the strain H37Rv reference genome. Two of the 147 original internal outbreak variants could not be lifted over to the strain H37Rv reference genome and therefore could not be identified by any tool combination when using the strain H37Rv genome, we therefore include only the 145 internal outbreak SNPs with respect to the strain H37Rv genome in our performance metrics.

From each tip genome, we simulated Illumina short-read sequence data, mapped reads and called variants as described above.

### Phylogenetic inference

To determine the effect of filtering on phylogenetic inference, we focused on variants identified by a single tool combination, BWA/GATK. We selected variant quality thresholds that corresponded to deciles of variant sites (including all sites across samples called as alternative alleles), generated multiple alignments of SNPs meeting quality thresholds, and inferred maximum-likelihood phylogenies for each multiple alignment. We fit maximum-likelihood trees with RAxML-ng, with a GTR substitution model. We applied a Stamatakis ascertainment bias correction to correct for invariant sites and specified nucleotide stationary frequencies present in the strain CDC1551 outbreak root genome. We measured phylogenetic distances from the best supported trees to the true tree using the Robinson–Foulds [55] and the Kendall–Colijn metrics [65], with lambda equal to 0. Bootstrap trees did not converge for all filtered alignments, reflecting the low levels of measured variation after filtering.

## Supplementary Data

Supplementary material 1
