# Supplementary material for: Genomic variant-identification methods may alter Mycobacterium tuberculosis transmission inferences
Source: Microb Genom. 2020 Jul 31;6(8):mgen000418. doi: 10.1099/mgen.0.000418 (PMC7641424; doi:10.1099/mgen.0.000418)
Supplement: Supplementary material 1 [file mgen-6-418-s001.pdf]

Supplementary Information.

Supplementary Text.

Transmission threshold adjustment. Many studies develop internal SNP thresholds, often using known epidemiological links to identify a maximum expected genetic distance between epidemiologically linked isolates, rather than applying externally developed SNP thresholds (1). To test if differences in transmission inferences between pipelines could be mitigated by adjusting thresholds to individual pipelines, we considered pairwise transmission predictions made after applying a 5-SNP threshold to pipeline E, the variants identified in the original study as "true" transmission links. We used the number of "true" transmission links to adjust SNP thresholds for every other pipeline to minimize the difference between the number of predicted transmission links to the "true" transmission links (Table S2). While adjusting distance thresholds so that pipelines predict similar numbers of transmission events does reduce observed differences between pipelines to some extent, pipeline differences remain substantial even after this adjustment (Fig. S5). For example, after applying adjusted SNP thresholds, the correlation of pairwise transmission links predicted by pipeline B correlated well with pipeline A (Cohen's kappa coefficient 0.80), but poorly with predictions from pipelines C, D, and E (Cohen's kappa coefficient 0.07, -0.04, and -0.19, respectively). However, adjusting SNP thresholds does improve the consistency of pipelines C and D (Cohen's kappa coefficient 0 before threshold adjustment and 0.66 following adjustment). Only pipelines A and B and pipelines C and D (Cohen's kappa coefficient 0.66) made similar transmission predictions, even after threshold adjustment.

Pairwise errors within repetitive genomic regions. Of 1283 total unique FP pairwise errors across all pipelines, 26.7% (354/1283) were identified by multiple mapping tools and 28.0% (359/1283) were identified by multiple variant calling tools. FP pairwise errors largely fell in repetitive regions: 47.4% (612/1283) of all FP pairwise errors occur in PE/PPE genes and 70.2% (901/1283) of all FP pairwise errors occurred in repeated regions identified with MUMmer (40). 70.6% (474/671) of the FP pairwise errors outside of PE/PPE genes occurred in the repeated regions identified by MUMmer. Further, FP pairwise errors cluster along the strain H37Rv genome. 63.7% of FP pairwise errors are within 5-bp of another FP error identified by the same combination of tools; 75.3% are within 10-bp.

We additionally investigated the source of persistent FP errors – errors outside PE/PPE genes that were not eliminated by filters – in two of the best-performing tool combinations, BWA/GATK/VQSR and Bowtie2/DeepVariant/QUAL. Five of 7 positions with persistent FP errors after VQSR occurred at positions that had failed a VQSR filter for other samples, though not the query samples, indicating that sites which fail VQSR for a single sample are potentially problematic sites across all samples. Bowtie2/DeepVariant/QUAL identified only a single FP pairwise error, even while including PE/PPE genes. This error occurred in samples where there was low (< 5X) coverage of sites that were called as the reference allele. However, the erroneous allele calls did not fail quality filters because no quality score was reported for reference allele calls.

We additionally investigated the genomic location of FN pairwise errors. The majority of FN pairwise errors are introduced through filtering (Fig. 4) and could potentially be reduced by adjusting filters. Among the 28 unique FN pairwise errors occurring prior to filtering (i.e. pairwise differences missed by tool combinations that could not be explained by filters), 60.7%

(17/28) are repeated by all three mapping tools and 71.4% (20/28) are repeated by all five variant identification tools. 21.4% (6/28) occurred in the PE/PPE genes and 28.6% (8/28) occurred in repeated regions identified with MUMmer(40). 27.2% (6/22) of the FN pairwise errors outside of PE/PPE genes occurred in the repeated regions identified by MUMmer. 17.3% of FN pairwise errors are within 5-bp of another FP error identified by the same combination of tools; 33.7 % are within 10-bp.

Ten of the 28 unique FN pairwise errors were repeated across all mappers and variants callers; all occurred outside of the PE/PPE genes. Six of these persistent FN pairwise errors were clustered within a ~100-bp region (2300100 – 2300200-bp), identified as a repetitive region by MUMmer. These 6 FN pairwise errors occurred in the gene *pks12* (Polyketide synthase), encoded by the largest open reading frame in the *M. tuberculosis* genome, comprised of a tandem repeat of two modules and previously identified as a potential recombination site (2).

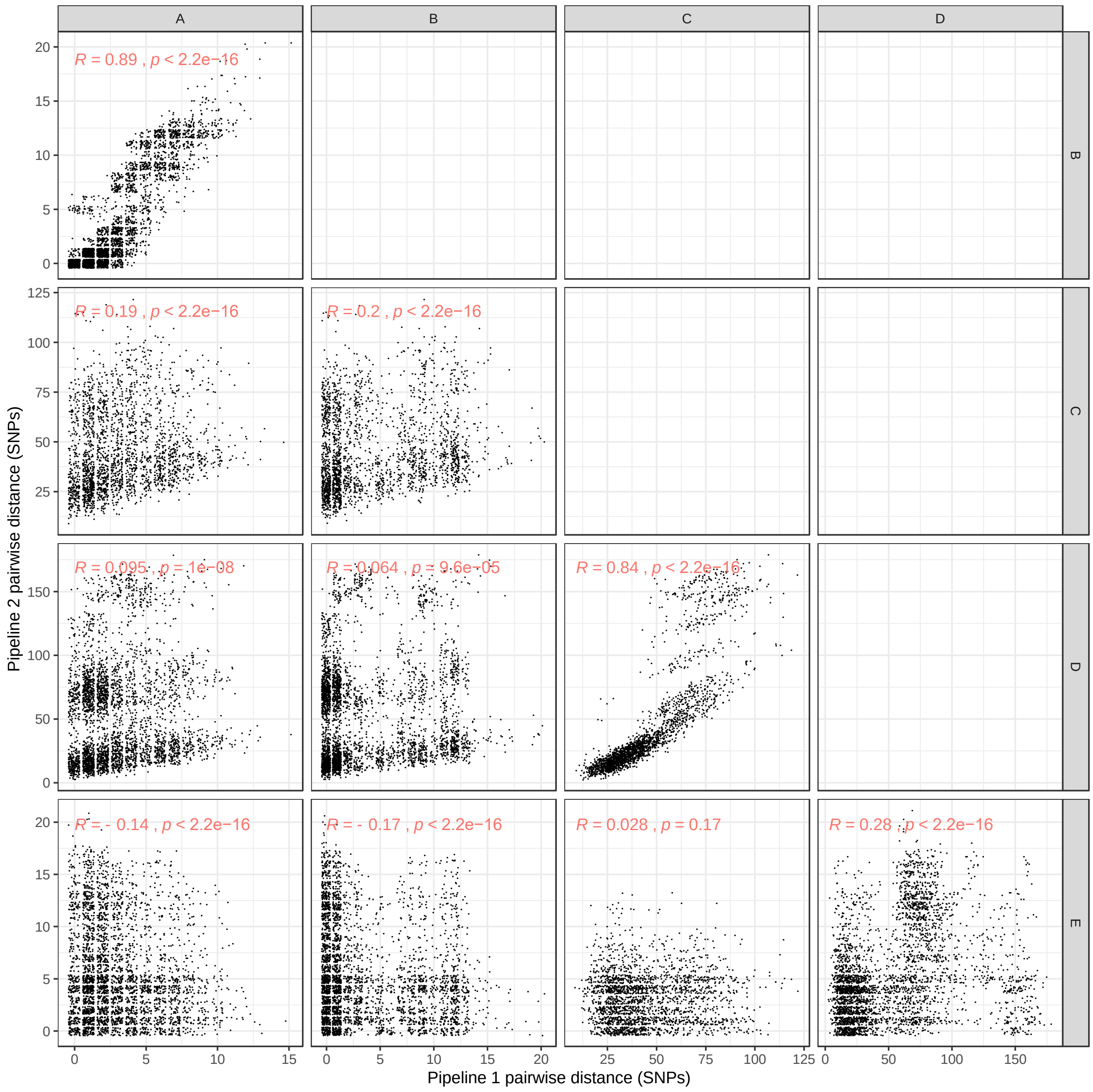

Figure S1. Pairwise SNP distances across variant calling pipelines. Each point corresponds to a unique pair of sequences and points are slightly jittered. Pearson's correlation coefficients are reported in red; x- and y-axes vary across the grid.

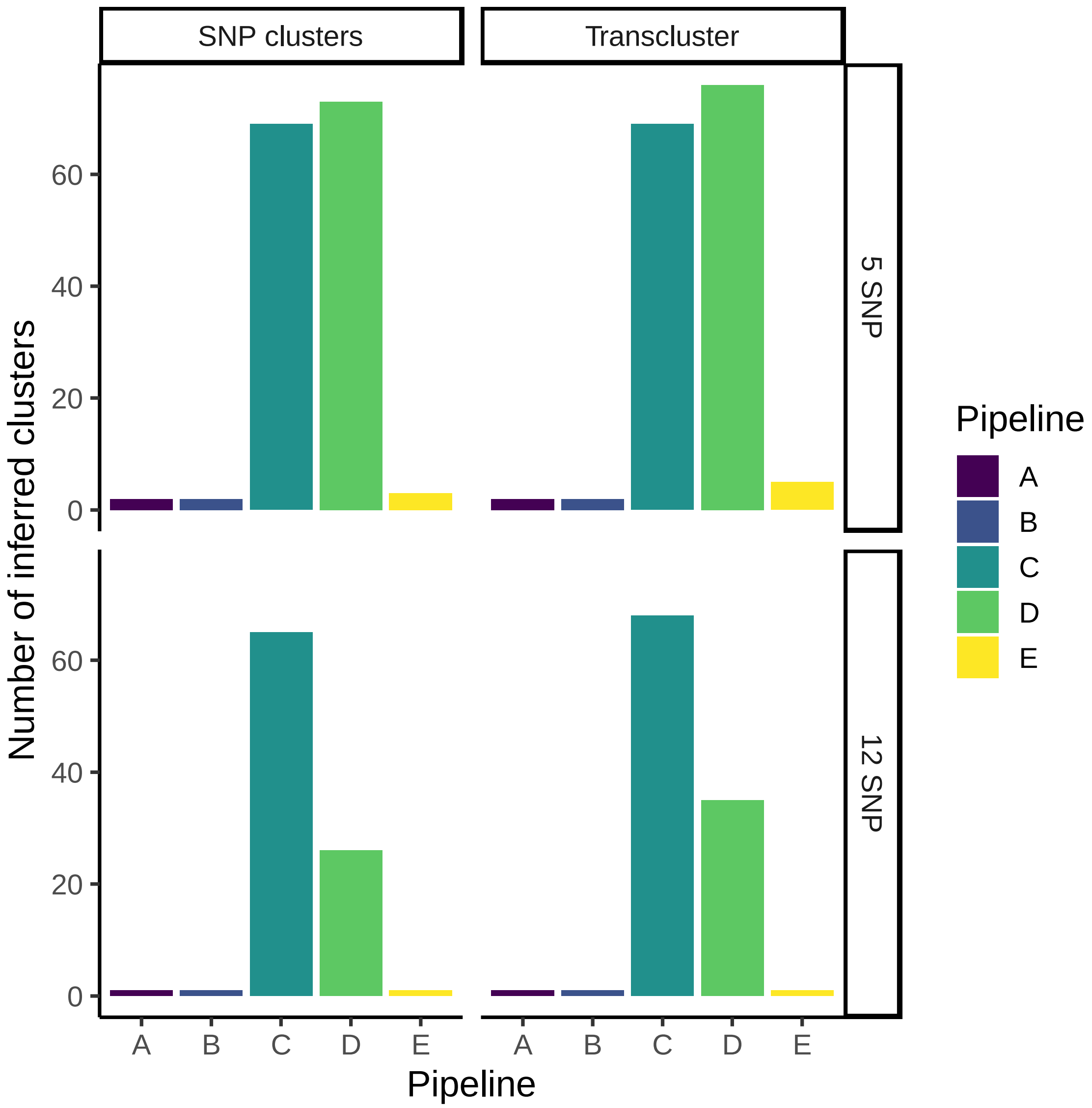

Figure S2. Transmission clusters inferred across variant calling pipelines. Transmission clusters were inferred from pairwise SNP distances (left column) or with *transcluster* (right column), which additionally incorporates epidemiological parameters and sampling dates. Both 5-SNP (top panel and 12-SNP thresholds (bottom panel) were applied to both methods.

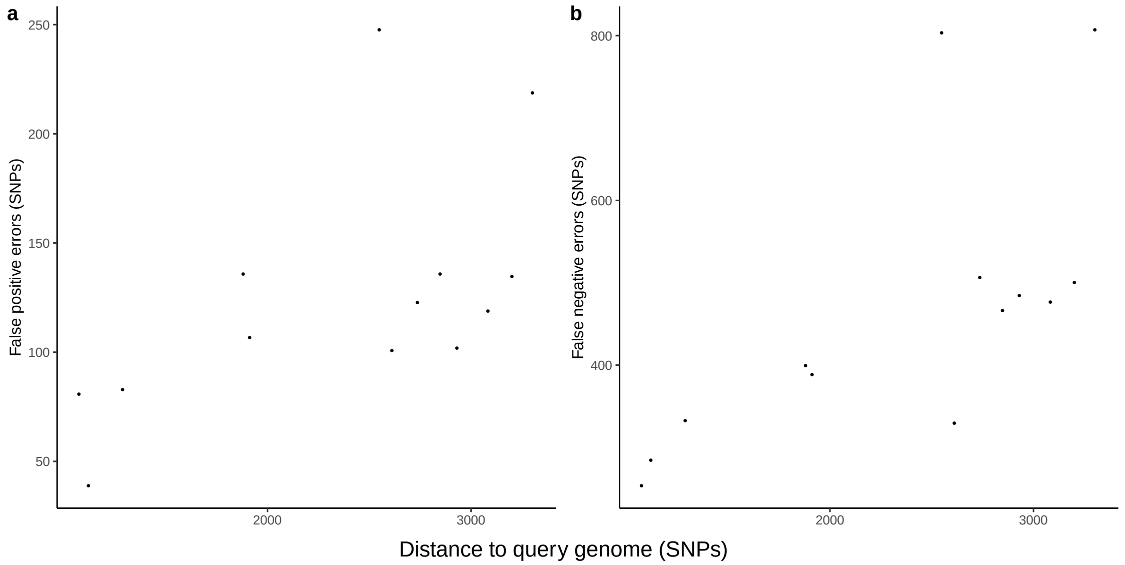

Figure S3. Errors increase with increasing distance between query and reference genomes. Prior to filtering, (a) false positive (Pearson’s r = 0.34, p-value < 0.001) and (b) false negative errors (r = 0.18, p-value = 0.02) increase with increasing distance between the reference and query genome, CDC1551 for a single tool combination (BWA/GATK). The x-axis is log-transformed and y-axes are on different scales.

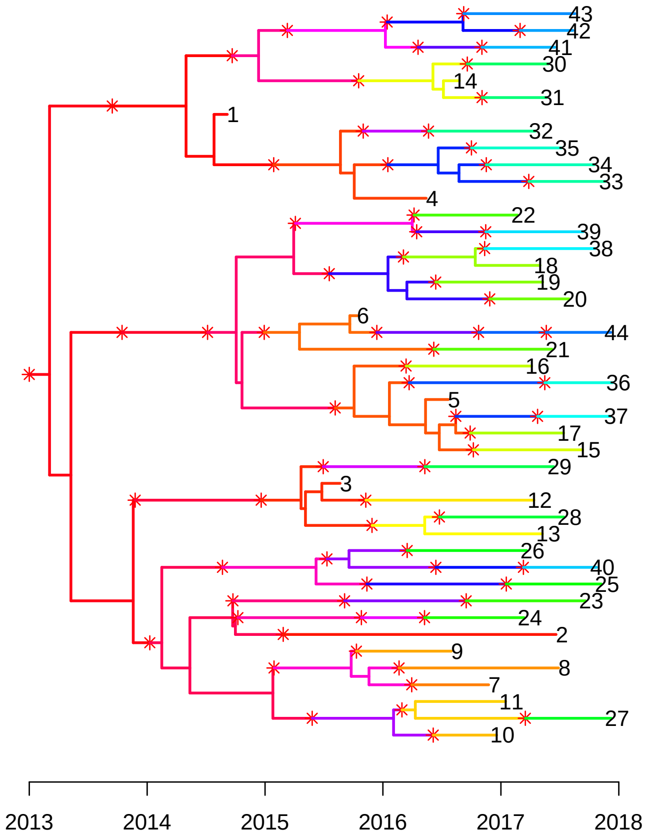

Figure S4. Simulated *M. tuberculosis* outbreak transmission tree. Transmission tree of a five-year clonal tuberculosis with a basic reproduction number, R_0, of 3 simulated with *TransPhylo* (3,4). The tree topology represents the underlying *M. tuberculosis* outbreak phylogeny. Branches are colored according to infected hosts. Stars represent transmission events. Numbered tips represent sampled isolates.

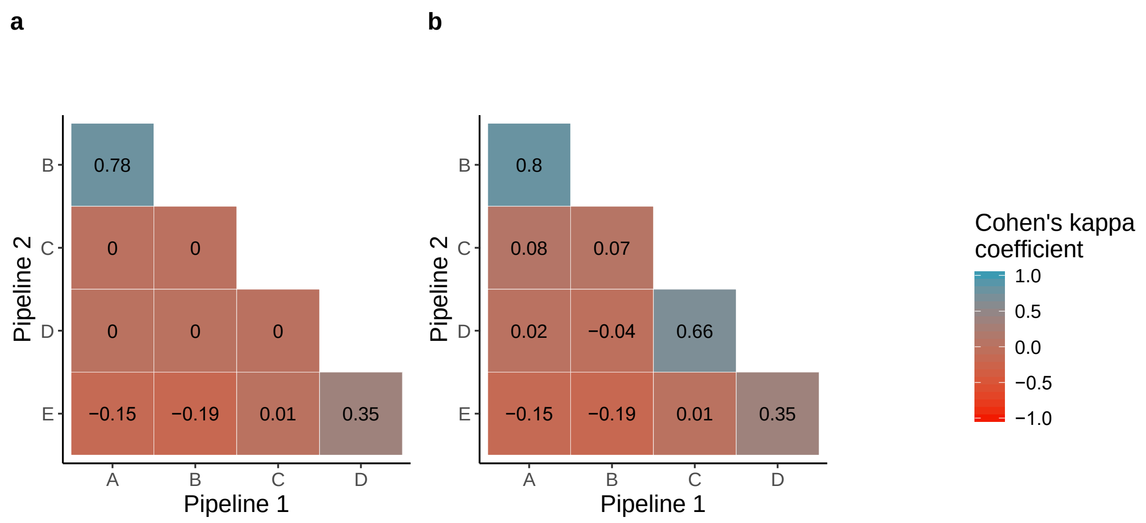

Figure S5. Consistency of transmission predictions. Consistency of pipelines in assigning potential transmission links measured by Cohen's kappa coefficient after applying (a) a SNP-distance threshold of 5 SNPs and (b) an SNP threshold adjusted to the specific pipeline. To adjust SNP thresholds for each pipeline, we considered pairwise transmission predictions made after applying a 5-SNP threshold to pipeline E, the variants identified in the original study, as "true" transmission links. We used the number of "true" transmission links to adjust SNP thresholds for every other pipeline to minimize the difference between the number of predicted transmission links to the "true" transmission links (Table S2). Cohen's kappa is a statistic used to measure inter-rater reliability that accounts for the probability two classifiers will make consistent predictions due to chance. Cohen's kappa of 1 indicates perfect agreement between classifiers and kappa less than 0 indicates no agreement.

Supplementary Table 1. *M. tuberculosis* variant calling pipelines compared in this study.

Pipeline	Source	Quality control	Reference Genome	Mapper	Caller	Ploidy	Variants output	Variant filters	Regions excluded
A		Read trimming with Sickle for reads with quality >20 and length > 30	*M. tuberculosis* common ancestor	Bowtie 2 v. 2.2.9	Samtools/bcftools v.1.3.1	diploid	Single sample VCFs, variant sites only, in addition to a multi-FASTA file.	Mapping Quality > 30; depth ≥ 10X; fixed mutations (with a frequency of ≥ 75%); strand bias filter option on	Excluded all SNPs that were located in repetitive regions of the genome (for example, PPE/PEPGRS family genes, phage sequence, insertion or mobile genetic elements) that are difficult to characterize with short-read sequencing technologies; small insertions or deletions identified by VarScan (version 2.3.9) also excluded
B	https://github.com/TGenNorth/NASP(5)		7199-99 NC_020089.1	BWA	GATK	haploid	Multi-sample, variant sites only.	10X threshold for depth of coverage and 0.9 proportion in base consensus	Masked any duplicated regions in the reference genome called by NUCmer
C	https://github.com/CPTR-ReSeqTB/UVP	FastQValidator, Kraken	H37Rv NC_000962.3	BWA	GATK	diploid	Single sample VCFs, variant sites only.	Base quality >=20; mapping quality >=20, depth >=10X; <= 3 SNPs within 10 bp region.	Excluded repeat and problematic loci
D		TrimGalore for reads with quality > 15 and stringency of 7	H37Rv NC_000962.2	BWA	Samtools/bcftools v.1.5	haploid	Single sample VCFs, variant sites only.	Filtered SNPs within 15-bp of indels; QUAL > 100; (DP4[2]+DP4[3])/(DP4[0]+DP4[1]+DP4[2]+DP4[3]) > 0.75	Excluded PE/PPE and other highly repetitive regions, plus 50 bp upstream and downstream of the same
E	(6)		H37Rv NC_000962.2	SARUMAN	Custom Perl script	haploid	Multi-sample FASTA, variant sites only.	Depth ≥ 10X, MAF ≥ 80%	Excluded 15 SNPs in repetitive regions such as PPE, PE_PGRS, ESX gene families that were false positives in Sanger sequencing

Supplementary Table 2. Summary of SNP variants identified by each pipeline. Pipeline, number of samples passing quality filters, total internal SNPs included in FASTA, sensitivity to Sanger-sequence confirmed SNPs, mean and median pairwise distances, percentage of identical pairwise comparisons, percentage of sequence pairs falling within the 5 and 12-SNP thresholds for potential transmission, updated SNP threshold, and percentage of sequence pairs falling within the 5 and 12-SNP thresholds for potential transmission. Internal SNPs refer to the number of SNPs in the VCF file that are variable within the outbreak. Pipeline C identifies SNPs in 69 of 86 samples; the updated threshold was selected for consistency with pipeline E in predictions for those 69 samples; hence the overall greater clustering.

Pipeline	Samples	SNPs	Sensitivity	Mean pairwise	Median pairwise	Identical (%)	<= 5 SNPs (%)	<= 12 SNPs (%)	Updated SNP threshold	<= Updated threshold (%)
A	86	94	92.9	3.1	2	11.1	80.7	99.9	3.0	65.2
B	86	68	72.9	3.8	1	29.7	69.8	96.5	3.0	64.4
C	69	352	88.2	45.5	40	0.0	0.0	0.2	71.0	86.4
D	86	416	90.6	54.0	42	0.0	0.4	8.0	66.0	63.4
E	86	85	100.0	5.5	4	9.6	63.1	89.2	5.0	63.1

Supplementary Table 3. Performance of pipelines for identifying SNP variants in the CDC1551 query genome using the H37Rv reference genome. Mapper, caller, filter, total true SNPs; SNPs reported by pipeline; TP, true positive; FP, false positive; FN, false negative; F1 score (harmonic mean of precision and sensitivity); recall, or sensitivity; and precision for each tool combination investigated. Filters include Raw, no filtering; Qual, filtering variants with a quality score of less than 40; and VQSR, filtering with GATK's Variant Quality Score Recalibration. Mean for 20 mutated query genomes.

Mapper	Caller	Filter	Truth	Reported	TP	FN	FP	F1	Recall	Precision	Per. Filtered
Bowtie 2	Breseq	Raw	1501	1066.5	1069.0	432.1	21.4	0.825	0.712	0.980	
Bowtie 2	Breseq	Qual	1501	1054.2	1062.8	438.2	16.4	0.824	0.708	0.984	1.2
Bowtie 2	DeepVariant	Raw	1501	1537.3	1185.8	315.1	351.4	0.781	0.790	0.772	
Bowtie 2	DeepVariant	Qual	1501	1071.5	1041.8	459.2	29.8	0.810	0.694	0.972	30.3
Bowtie 2	GATK	Raw	1501	1253.0	1164.2	336.8	101.5	0.841	0.776	0.919	
Bowtie 2	GATK	Qual	1501	1250.0	1150.5	350.5	99.5	0.836	0.766	0.920	0.2
Bowtie 2	GATK	VQSR	1501	1033.8	1025.8	475.2	8.1	0.809	0.683	0.992	17.5
Bowtie 2	Pilon	Raw	1501	1391.4	1201.9	299.1	191.1	0.831	0.801	0.863	
Bowtie 2	Pilon	Pilon	1501	1297.8	1175.3	325.6	124.0	0.839	0.783	0.904	6.7
Bowtie 2	Pilon	Qual	1501	1369.7	1196.5	304.4	173.5	0.834	0.797	0.873	1.6
Bowtie 2	Samtools	Raw	1501	1425.0	1199.7	301.4	225.4	0.820	0.799	0.842	
Bowtie 2	Samtools	Qual	1501	1346.9	1184.0	316.9	162.8	0.832	0.789	0.879	5.5
Bowtie 2	Samtools	VQSR	1501	1271.5	1149.4	351.6	122.2	0.829	0.766	0.905	10.8
BWA	DeepVariant	Raw	1501	1254.7	1150.2	350.9	104.5	0.835	0.766	0.917	
BWA	DeepVariant	Qual	1501	1084.0	1056.2	444.9	27.8	0.817	0.704	0.974	13.6
BWA	GATK	Raw	1501	1238.9	1169.4	331.6	83.5	0.849	0.779	0.933	
BWA	GATK	Qual	1501	1236.8	1153.7	347.4	83.2	0.843	0.769	0.933	0.2
BWA	GATK	VQSR	1501	1062.1	1051.3	449.6	10.8	0.820	0.700	0.990	14.3
BWA	Pilon	Raw	1501	1246.4	1146.0	354.9	100.3	0.834	0.764	0.919	
BWA	Pilon	Pilon	1501	1234.2	1139.0	361.9	95.2	0.833	0.759	0.923	1.0
BWA	Pilon	Qual	1501	1242.1	1145.8	355.2	96.3	0.835	0.763	0.922	0.3
BWA	Samtools	Raw	1501	1273.2	1155.7	345.3	117.5	0.833	0.770	0.908	
BWA	Samtools	Qual	1501	1248.5	1145.5	355.5	103.0	0.833	0.763	0.918	1.9
BWA	Samtools	VQSR	1501	1180.2	1114.0	387.1	66.2	0.831	0.742	0.944	7.3
SMALT	DeepVariant	Raw	1501	1349.2	1175.7	325.3	173.5	0.825	0.783	0.871	
SMALT	DeepVariant	Qual	1501	1095.9	1063.1	437.9	32.8	0.819	0.708	0.970	18.8
SMALT	GATK	Raw	1501	1277.7	1183.8	317.2	108.2	0.847	0.789	0.915	
SMALT	GATK	Qual	1501	1276.2	1167.9	333.1	108.3	0.841	0.778	0.915	0.1
SMALT	GATK	VQSR	1501	1054.9	1042.6	458.4	12.3	0.816	0.695	0.988	17.4
SMALT	Pilon	Raw	1501	1358.8	1186.1	314.9	172.7	0.830	0.790	0.873	
SMALT	Pilon	Pilon	1501	1290.5	1167.3	333.6	123.2	0.836	0.778	0.905	5.0
SMALT	Pilon	Qual	1501	1346.9	1184.0	317.1	162.9	0.831	0.789	0.879	0.9
SMALT	Samtools	Raw	1501	1352.8	1186.0	315.0	166.8	0.831	0.790	0.877	
SMALT	Samtools	Qual	1501	1318.0	1174.5	326.5	143.6	0.833	0.782	0.891	2.6
SMALT	Samtools	VQSR	1501	1251.8	1136.2	364.9	115.6	0.825	0.757	0.908	7.5

Supplementary Table 4. Reference genomes used for mapping. SNP distance to query are those SNPs identified by pairwise alignment of CDC1551 with the given reference, using the parameters described in the Methods.

**Strain**	**GenBank assembly accession**	**Lineage**	**Distance (SNPs) to Query Genome, CDC1551**
CDC1551	GCA_000008585.1	4	0
H37Rv	GCA_000195955.2	4	1501
BCG Pasteur 1173P2	GCA_000009445.1	*M. bovis*	3108
F11	GCA_000016925.1	4	1376
KZN_1435	GCA_000023625.1	4	1402
Beijing_NITR203	GCA_000364825.1	2	3396
HN-024	GCA_002356255.1	1	2700
18b	GCA_000835125.1	2	1908
GM041182	GCA_000253355.1	6	2921
96121	GCA_000756545.1	1	2825
EAI5/NITR206	GCA_000389945.1	1	3260
EAI5	GCA_000422125.1	1	2566
W-148	GCA_000193185.2	2	2502
26105	GCA_001545055.1	3	1933

Supplementary Table 5. Performance of pipelines for identifying pairwise differences in outbreak genomes. Mapper, caller, filter, total true SNPs; SNPs reported by pipeline; TP, true positives; FP, false positive; FN, false negative; F1 score (harmonic mean of precision and sensitivity); recall, or sensitivity; precision; and percentage of variants filtered, the percentage of total allele calls at candidate outbreak SNP positions (including invariant and variant allele calls) excluded by filters. Filters include Raw, no filtering; Qual, filtering variants with a quality score of less than 40; Pilon, Pilon specific filters; and VQSR, filtering with GATK's Variant Quality Score Recalibration. All statistics are the mean of all pairwise comparisons. The true pairwise differences (mean 13.164) reflect the mean pairwise differences with respect to the H37Rv reference genome.

Mapper	Caller	Filter	Truth	Reported	TP	FP	FN	F1	Recall	Precision	Per. Filtered
Bowtie 2	Breseq	Raw	13.164	18.851	11.248	7.603	1.915	0.698	0.859	0.588	
Bowtie 2	Breseq	Qual	13.164	16.732	11.248	5.483	1.915	0.747	0.859	0.660	0.73
Bowtie 2	DeepVariant	Raw	13.164	50.906	12.510	38.396	0.654	0.395	0.950	0.250	
Bowtie 2	DeepVariant	Qual	13.164	11.321	11.319	0.002	1.845	0.926	0.862	1.000	18.43
Bowtie 2	GATK	Raw	13.164	21.598	12.439	9.160	0.725	0.715	0.943	0.576	
Bowtie 2	GATK	Qual	13.164	20.351	12.439	7.912	0.725	0.743	0.943	0.613	0.26
Bowtie 2	GATK	VQSR	13.164	12.869	11.897	0.971	1.266	0.918	0.900	0.937	16.88
Bowtie 2	Pilon	Raw	13.164	54.892	12.429	42.463	0.735	0.376	0.941	0.235	
Bowtie 2	Pilon	Pilon	13.164	13.059	12.429	0.630	0.735	0.946	0.941	0.950	17.73
Bowtie 2	Pilon	Qual	13.164	18.727	12.429	6.298	0.735	0.778	0.941	0.664	7.83
Bowtie 2	Samtools	Raw	13.164	88.964	12.388	76.576	0.776	0.255	0.941	0.147	
Bowtie 2	Samtools	Qual	13.164	32.465	12.230	20.235	0.933	0.546	0.928	0.387	4.81
Bowtie 2	Samtools	VQSR	13.164	41.867	12.241	29.626	0.923	0.461	0.930	0.306	8.25
BWA	DeepVariant	Raw	13.164	21.395	12.485	8.910	0.679	0.725	0.948	0.587	
BWA	DeepVariant	Qual	13.164	11.624	11.433	0.190	1.730	0.924	0.872	0.983	10.91
BWA	GATK	Raw	13.164	23.777	12.433	11.344	0.730	0.706	0.942	0.565	
BWA	GATK	Qual	13.164	23.217	12.431	10.785	0.733	0.717	0.942	0.579	0.22
BWA	GATK	VQSR	13.164	13.202	12.295	0.907	0.869	0.937	0.930	0.944	12.87
BWA	Pilon	Raw	13.164	19.868	11.928	7.940	1.236	0.719	0.907	0.596	
BWA	Pilon	Pilon	13.164	12.356	11.928	0.428	1.236	0.937	0.907	0.969	7.44
BWA	Pilon	Qual	13.164	12.463	11.928	0.535	1.236	0.931	0.907	0.956	3.16
BWA	Samtools	Raw	13.164	34.965	12.164	22.801	1.000	0.509	0.924	0.351	
BWA	Samtools	Qual	13.164	19.968	12.112	7.856	1.052	0.739	0.920	0.617	1.74
BWA	Samtools	VQSR	13.164	19.221	12.134	7.087	1.030	0.756	0.922	0.640	7.15
SMALT	DeepVariant	Raw	13.164	29.938	12.505	17.432	0.659	0.589	0.949	0.427	
SMALT	DeepVariant	Qual	13.164	11.577	11.433	0.144	1.730	0.926	0.872	0.987	12.28
SMALT	GATK	Raw	13.164	19.941	12.425	7.516	0.739	0.766	0.942	0.645	
SMALT	GATK	Qual	13.164	19.349	12.425	6.924	0.739	0.778	0.942	0.662	0.14
SMALT	GATK	VQSR	13.164	13.506	12.133	1.373	1.031	0.913	0.918	0.907	16.97
SMALT	Pilon	Raw	13.164	57.636	11.928	45.708	1.236	0.368	0.907	0.231	
SMALT	Pilon	Pilon	13.164	12.373	11.928	0.445	1.236	0.936	0.907	0.966	17.33
SMALT	Pilon	Qual	13.164	16.304	11.928	4.376	1.236	0.847	0.907	0.794	9.32
SMALT	Samtools	Raw	13.164	44.951	12.237	32.715	0.927	0.427	0.928	0.277	
SMALT	Samtools	Qual	13.164	23.221	12.237	10.984	0.927	0.677	0.928	0.532	2.19
SMALT	Samtools	VQSR	13.164	23.202	12.230	10.971	0.933	0.684	0.927	0.542	7.26

Supplementary Table 6. Variant calling tools and versions used in the comparison of variant calling tools.

Software	Source	Version	Use
Bowtie2(7)	http://bowtie-bio.sourceforge.net/bowtie2/index.shtml	2.3.4.2	Maps reads
BWA(8)	http://bio-bwa.sourceforge.net/	0.7.15-r1140	Maps reads
SMALT(9)	https://www.sanger.ac.uk/science/tools/smalt-0	0.7.6	Maps reads
Sambamba	https://lomereiter.github.io/sambamba/	0.6.6	Post-process BAM files, mark duplicates
Bcftools/Samtools (10)	https://samtools.github.io/bcftools/	1.9-105-gaf6f0c9	Variant calling/VCF processing
GATK (11)	https://software.broadinstitute.org/gatk/	4.1.0.0	Variant calling
DeepVariant (12)	https://github.com/google/deepvariant	0.7.0	Variant calling
Breseq (13)	https://github.com/barricklab/breseq/releases/tag/v0.34.1	0.34.1	Variant calling
Pilon (14)	https://github.com/broadinstitute/pilon	1.23	Variant calling
hap.py (15)	https://github.com/Illumina/hap.py	3.10	Measures performance
MUMmer (16)	http://mummer.sourceforge.net/	3.1	Pairwise alignment
RAxML-ng (17)	https://github.com/amkozlov/raxml-ng	0.8.1	Fit maximum likelihood trees
ART (18)	https://www.niehs.nih.gov/research/resources/software/biostatistics/art/index.cfm	2.5.8	Sequence read simulation

1. Hatherell HA, Colijn C, Stagg HR, Jackson C, Winter JR, Abubakar I. Interpreting whole genome sequencing for investigating tuberculosis transmission: A systematic review. BMC Med. 2016;14(1):1–13.
2. Phelan JE, Coll F, Bergval I, Anthony RM, Warren R, Sampson SL, et al. Recombination in pe/ppe genes contributes to genetic variation in Mycobacterium tuberculosis lineages. BMC Genomics. 2016;17(1):1–12.
3. Didelot X, Fraser C, Gardy J, Colijn C, Malik H. Genomic infectious disease epidemiology in partially sampled and ongoing outbreaks. Mol Biol Evol. 2017;34(4):997–1007.
4. Didelot X, Gardy J, Colijn C. Bayesian inference of infectious disease transmission from whole-genome sequence data. Mol Biol Evol. 2014;31(7):1869–79.
5. Sahl JW, Lemmer D, Travis J, Schupp JM, Gillece JD, Aziz M, et al. NASP: an accurate, rapid method for the identification of SNPs in WGS datasets that supports flexible input and output formats. Microb genomics. 2016 Aug 1;2(8):e000074.
6. Roetzer A, Diel R, Kohl TA, Rückert C, Nübel U, Blom J, et al. Whole Genome Sequencing versus Traditional Genotyping for Investigation of a Mycobacterium tuberculosis Outbreak: A Longitudinal Molecular Epidemiological Study. PLoS Med. 2013;10(2).
7. Langmead B, Salzberg SL. Fast gapped-read alignment with Bowtie 2. Nat Methods. 2012 Apr;9(4):357–9.
8. Li H, Durbin R. Fast and accurate short read alignment with Burrows-Wheeler transform. Bioinformatics. 2009 Jul 15;25(14):1754–60.
9. Genome Research Ltd. SMALT. 2015.
10. Li H. A statistical framework for SNP calling, mutation discovery, association mapping and population genetical parameter estimation from sequencing data. Bioinformatics. 2011 Nov 1;27(21):2987–93.
11. Van der Auwera GA, Carneiro MO, Hartl C, Poplin R, del Angel G, Levy-Moonshine A, et al. From FastQ Data to High-Confidence Variant Calls: The Genome Analysis Toolkit Best Practices Pipeline. In: Current Protocols in Bioinformatics. Hoboken, NJ, USA: John Wiley & Sons, Inc.; 2013. p. 11.10.1-11.10.33.
12. Poplin R, Chang PC, Alexander D, Schwartz S, Colthurst T, Ku A, et al. A universal SNP and small-indel variant caller using deep neural networks. Vol. 36, Nature Biotechnology. Nature Publishing Group; 2018. p. 983.
13. Deatherage DE, Barrick JE. Identification of mutations in laboratory-evolved microbes from next-generation sequencing data using breseq. Methods Mol Biol. 2014;1151:165–88.
14. Walker BJ, Abeel T, Shea T, Priest M, Abouelliel A, Sakthikumar S, et al. Pilon: An integrated tool for comprehensive microbial variant detection and genome assembly improvement. Wang J, editor. PLoS One. 2014 Nov 19;9(11):e112963.
15. Krusche P. Haplotype VCF comparison tools. Illumina; 2019.
16. Kurtz S, Phillippy A, Delcher AL, Smoot M, Shumway M, Antonescu C, et al. Versatile and open software for comparing large genomes. Genome Biol. 2004;5(2):R12.
17. Kozlov AM, Darriba D, Flouri T, Morel B, Stamatakis A. RAxML-NG: A fast, scalable, and user-friendly tool for maximum likelihood phylogenetic inference. Wren J, editor. Bioinformatics. 2019 May 9;

18. Huang W, Li L, Myers JR, Marth GT. ART: a next-generation sequencing read simulator. Bioinformatics. 2012 Feb 15;28(4):593–4.
